# Rice Ribosomal Protein Large Subunit Genes and Their Spatio-temporal and Stress Regulation

**DOI:** 10.3389/fpls.2016.01284

**Published:** 2016-08-24

**Authors:** Mazahar Moin, Achala Bakshi, Anusree Saha, Mouboni Dutta, Sheshu M. Madhav, P. B. Kirti

**Affiliations:** ^1^Department of Plant Sciences, University of HyderabadHyderabad, India; ^2^Department of Biotechnology, Indian Institute of Rice ResearchHyderabad, India

**Keywords:** ribosomal proteins, abiotic stress, biotic stress, gene expression, rice

## Abstract

Ribosomal proteins (RPs) are well-known for their role in mediating protein synthesis and maintaining the stability of the ribosomal complex, which includes small and large subunits. In the present investigation, in a genome-wide survey, we predicted that the large subunit of rice ribosomes is encoded by at least 123 genes including individual gene copies, distributed throughout the 12 chromosomes. We selected 34 candidate genes, each having 2–3 identical copies, for a detailed characterization of their gene structures, protein properties, *cis*-regulatory elements and comprehensive expression analysis. RPL proteins appear to be involved in interactions with other RP and non-RP proteins and their encoded RNAs have a higher content of alpha-helices in their predicted secondary structures. The majority of RPs have binding sites for metal and non-metal ligands. Native expression profiling of 34 ribosomal protein large (RPL) subunit genes in tissues covering the major stages of rice growth shows that they are predominantly expressed in vegetative tissues and seedlings followed by meiotically active tissues like flowers. The putative promoter regions of these genes also carry *cis*-elements that respond specifically to stress and signaling molecules. All the 34 genes responded differentially to the abiotic stress treatments. Phytohormone and cold treatments induced significant up-regulation of several RPL genes, while heat and H_2_O_2_ treatments down-regulated a majority of them. Furthermore, infection with a bacterial pathogen, *Xanthomonas oryzae*, which causes leaf blight also induced the expression of 80% of the RPL genes in leaves. Although the expression of RPL genes was detected in all the tissues studied, they are highly responsive to stress and signaling molecules indicating that their encoded proteins appear to have roles in stress amelioration besides house-keeping. This shows that the RPL gene family is a valuable resource for manipulation of stress tolerance in rice and other crops, which may be achieved by overexpressing and raising independent transgenic plants carrying the genes that became up-regulated significantly and instantaneously.

## Introduction

Ribosomes are tiny (200–300Å) ribonucleoprotein complexes typically existing as two unequal sized subunits in all organisms and constituting 25–30% of total cell mass (Alberts et al., 2002). The ribosome complex, as a whole, performs mRNA-directed protein synthesis. Specific interaction of RPs and rRNA with mRNA, tRNA, and other non-ribosomal protein cofactors ensure that the process of initiation of protein synthesis, amino acid assembly and termination occurs appropriately in the cells (Maguire and Zimmermann, 2001). Eukaryotic ribosomes have a sedimentation coefficient of 80S with the large 60S subunit having 25S, 5.8S, 5S rRNA, and the small 40S subunit consisting of 18S rRNA (Ben-Shem et al., 2010). The number of RPs in ribosomes varies between organisms, with eukaryotes having up to 80 RPs and prokaryotes possess a total of only 54 RPs in both the subunits (Doudna and Rath, 2002).

The ribosomal gene family has more than 200 genes, but less than 100 corresponding RPs are incorporated into the ribosomes in all organisms including yeast, animals, and plants (Ban et al., 2000; Barakat et al., 2001; Hanson et al., 2004). This supports the fact that each RP-gene exists as 2–5 identical members with 95–100% nucleotide and predicted protein similarity. An RP synthesized from only one gene copy of a group incorporates into a ribosome under a given condition/tissue (Guarinos et al., 2003; Schuwirth et al., 2005). For example, the *Arabidopsis* genome has 249 genes for 80 RPs (48-large subunit proteins, 32-small subunit proteins) with each gene having 3–4 expressed copies and none exists as a single gene copy (Wool et al., 1996). RPs, in addition to their universal roles of stabilizing the ribosomal complex and mediating polypeptide synthesis also have extra-ribosomal functions such as their involvement in response to the environmental stresses (Warner and McIntosh, 2009; Sormani et al., 2011).

Mutations in plant RP genes have been implicated in perturbed phenotypes as has been seen in animal systems including humans. Earlier studies with *Arabidopsis* showed that mutations in many RP genes (*RPS18A, RPL24B, RPS5B, RPS13B*, and *RPL27A*) resulted in a ‘pointed first leaf’ phenotype characterized by reduced cell division and growth, and genotoxic sensitive plants (Lijsebettens et al., 1994; Revenkova et al., 1999; Ito et al., 2000; Szakonyi and Byrne, 2011). A T-DNA insertion mutation in the *Arabidopsis AtRPL10* gene caused lethal female gametophytes, while overexpression complemented the same with the recovery of the severe dwarf phenotype that resulted from the disruption of the *ACL5* gene (Imai et al., 2008). A transposon insertion mutation in one of the three copies of the *AtRPS13A* gene resulted in reduced cell division, late flowering, retarded root and leaf growth (Ito et al., 2000). Similar effects of plant growth retardation and reduced fertility were observed after knockdown of *AtRPL23aA* resulting in reduced synthesis of the RPL23aA protein, while knockout of its paralog, *RPL23aB*, had no effect on growth (Degenhardt and Bonham-Smith, 2008a). *RPL23aB* is the only RP paralog that did not produce any visible phenotypic defects upon knockout (Degenhardt and Bonham-Smith, 2008b).

These RP-gene knockout studies clearly show that although RP genes exist as multiple gene copies, the maximum possible expression of all the gene copies is required for them to be incorporated into the ribosomes during specific stages of growth and development and under certain stress conditions (Schmid et al., 2005; Byrne, 2009). The variation in the composition of ribosomes by the incorporation of RPs derived from identical members could be a major factor in the translational regulation of transcripts in different cell types and under various specific conditions (Giavalisco et al., 2005; Carroll et al., 2008). The change in the composition of RPs upon feeding of *Arabidopsis* leaves with sucrose further supports the heterogeneity of ribosomes in response to external stimuli (Hummel et al., 2012).

The expression of RP genes has also been shown to be differentially regulated by signaling molecules and environmental stresses. The transcript levels of *Arabidopsis RPS15a* (*RPS15aA*, *C*, *D* and *F*) were up-regulated in response to phytohormone and heat treatments (Hulm et al., 2005). Similar transcript abundance under BAP treatment was detected for *Arabidopsis RPS14*, *RPL13*, and *RPL30* genes (Cherepneva et al., 2003). Low temperature induced the expression of three RP genes; *RPS6*, *RPS13*, and *RPL37* in soybean (Kim et al., 2004) and a homolog of RPL13, *BnC24* in Brassica and *E. coli* (Sáez-Vásquez et al., 2000; Tanaka et al., 2001). The overexpression of RPL13 also resulted in tolerance against a fungal pathogen, *Verticillium dahliae* in transgenic potato with coordinated up-regulation of genes coding for defense and antioxidant enzymes (Yang et al., 2013) implying that RPs function in stress-response/tolerance through a network of multiple stress-related genes. In maize and *Arabidopsis*, RPL10A and RPL10C were shown to be significantly up-regulated under UV-B stress (Casati and Virginia, 2003; Ferreyra et al., 2010, 2013). *RPL44* was found to be up-regulated under osmotic stresses, and the overexpression of *Aspergillus glaucus RPL44* in yeast and tobacco ensured increased tolerance to salt and drought stresses (Liu et al., 2014). The majority of studies on RPs were undertaken in *Arabidopsis* largely because of the availability of insertion mutant lines and smaller genome size.

Until now, not much emphasis has been placed on the differential expression patterns of RP genes of rice in response to external stimuli. We had generated a large-scale enhancer based activation-tagged gain-of-function mutant population in *indica* rice, which was screened for water-use efficiency. Among the potential mutants with sustained productivity under prolonged water-limiting conditions, two of them were found to have enhanced expression of large subunit ribosomal genes because of their being tagged by the enhancers (Moin et al., 2016). This has prompted us to investigate the other rice RPL genes in the context of stress-responsiveness.

In the present study, we describe the genome-wide organization of predicted 123 RPL genes in rice including the individual gene copies. We investigated their overall expression pattern in selected tissues covering the major growth stages of rice. Also, we have provided an overview of their differential expression pattern under biotic and abiotic stress conditions that limit rice productivity. We identified specific RP genes, whose expression is unique or overlapping under native and treated conditions. In summary, the information presented in this study provides a resource for subsequent exploitation of RPL genes to ameliorate abiotic and biotic stress conditions in rice and also other crop plants in future.

## Materials and Methods

### Nucleotide Sequence Retrieval of RPL Genes

To identify the total members of the large subunit ribosomal gene family, a keyword search using “ribosomal” was performed under the putative function search of Rice Genome Annotation Project Data Base (RGAP-DB v7)^1^ and Phytozome v11^2^. The large subunit members were shortlisted by selecting the genes starting with prefix ‘L,’ for large subunit as opposed to ‘S’ that specifies small subunit genes. A total of 123 RPL genes were identified, and since the number of RPL genes in both the databases was same, the gene sequences were downloaded from RGAP-DB. When further looked for the presence of identical members or copies of each gene in RGAP-DB, we observed that each RPL gene has an average of 2–3 gene copies in the genome. From these 123 genes, we selected 34 candidate genes each representing one orthologous group excluding the identical copies for expression studies. All the identified 123 sequences were also confirmed through nucleotide and protein BLAST search in the NCBI^3^ and Hidden Markov Model (HMM) of Pfam^4^ databases, respectively. The predicted protein sequences of all the 123 RPL genes were also verified in NCBI conserved domain database^5^. To minimize the missing of potential RPL genes and to ensure that all the identified sequences belong to the ribosomal large subunit gene family, multiple databases were employed.

### Chromosomal Distribution of RPL Genes

To determine the chromosomal distribution, the locus number of each of the 123 RPL genes obtained from RGAP-DB was submitted to the OryGenesDB^6^. Based on the output generated in OryGenesDB, the position of each gene at its corresponding locus on the chromosome was located manually.

### RPL Gene Structures

The structure of each of the 34 RPL genes was determined to study the number and position of introns and exons, GC-content, gene orientation in the genome and alternative splice forms. The full-length sequences of each gene and cDNA were submitted to the Gene Structure Display Server (GSDSv2)^7^ to predict the structure.

### Protein Properties, Secondary Structure, Homology Modeling, and Phylogenetic Analysis

The predicted sequences of 34 RPL proteins were obtained from the RGAP-DB and analyzed using an online tool, PSORT^8^ to predict the protein properties such as size, molecular weight and isoelectric point (*p*I). The amino acid sequences of these proteins were aligned in ClustalW^9^ and submitted to the Molecular Evolutionary Genetic Analysis (MEGAv6)^10^ program for constructing an unrooted phylogenetic tree to identify the protein similarities in the RPL family in rice. The domains and motifs in proteins were identified using SMART^11^ (Simple Modular Architecture Research Tool). The GRAVY (Grand average of hydropathicity) indices of RPL proteins, which are the determinants of the hydrophobicity of whole protein was calculated using ExPASy ProtParam^12^. The GRAVY values of most of the proteins are usually in the range of +2 to -2, and values in negative range or less than zero indicate that the proteins are hydrophilic in nature (Song et al., 2015).

Although the detailed crystal structure of ribosomal complex has been well-characterized (Ben-Shem et al., 2010), we tried to study the properties of individual RPs. To gain an insight into the secondary structure of RPL proteins and to characterize the presence of metal–ligand/protein/RNA interacting sites, the three-dimensional secondary structures of 34 RPL proteins were predicted using Phyre2^13^ program (Protein Homology/AnalogY Recognition Engine v2; Kelley et al., 2015). Individual protein sequences were submitted in Phyre2 in FASTA format and after studying the properties such as α-helices and β-strands, they were directed to 3DLigandSite^14^ (Wass et al., 2010) to predict the metal/non-metal ligands and their binding sites in each protein.

### *In silico* Putative Promoter Analysis of 34 RPL Genes

To determine the presence of stress-responsive *cis*-regulatory elements, the nucleotide sequence ≤1 kb upstream of each RPL gene was retrieved from RGAP-DB and submitted to the Plant *Cis*-Acting Regulatory Elements^15^ database. The location and number of repeats of each *cis*-regulatory sequence in the putative promoter regions of each RPL gene were identified.

### Plant Material and Growth Conditions

The seeds of *Oryza sativa* L. sp. *indica* var. Samba Mahsuri (BPT-5204) maintained in greenhouse conditions were surface sterilized with 70% ethanol for 50–60 s followed by 4% sodium hypochlorite for 20 min. Seeds were then washed thrice with sterile double-distilled water, blot dried and cultured on solid MS medium at 28 ± 2°C and 16 h light/8 h dark photoperiods.

To analyze the native tissue-specific expression pattern of 34 RPL genes at different stages of rice development, samples were collected from 13 different tissues covering major stages of rice growth. After sterilization, seeds were soaked in water in a rotary shaker and after 16 h of incubation, the embryonic portion was manually cut under a stereo-microscope to collect the embryos and endosperm. Some of these seeds were allowed to continue to germinate on MS medium. After 3 and 6 days of germination, the plumules, radicles, shoot, and leaf tissues were collected separately. After 2 weeks of growth on MS medium, some of the seedlings were transferred to pots containing alluvial soil and grown under greenhouse conditions (30 ± 2°C, 16 h light/8 h dark photoperiods). Plants were amply watered with RO (Reverse Osmosis) purified water up to 3 cm overlay in the pots as required for normal growth of rice. About 45 days after transfer (DAT) to the greenhouse, rice plants were uprooted to collect shoot, root, flag leaf, and root–shoot transition tissues. After 60 DAT, flowers, partially filled grains and spikes were collected.

### Abiotic Stress Treatments to Seedlings

To analyze the differential expression pattern of 34 RPL genes and to distinguish RPL genes that are up/down-regulated under abiotic conditions, 7-day-old seedlings were exposed to five different abiotic treatments such as MeJa, SA, cold stress (4°C), heat stress (42°C), and oxidative stress (H_2_O_2_). The 7-d-old seedlings were dipped in the solutions of 100 μM MeJa (Wang et al., 2007), 3 mM SA (Mitsuhara et al., 2008), and 10 μM H_2_O_2_ (Fuller, 2007). The root and shoot tissues were collected separately at 5 min, 3 h, 6 h, 24 h, and 60 h after treatments. For cold and heat induced stresses, seedlings in water were exposed to 4°C and 42°C (Jami et al., 2012), respectively, and root and shoot samples were collected at time intervals as described. Since WT rice seedlings started to wilt after 24 h of exposure to 42°C, heat stress samples were collected up to 24 h only. Seedlings in water at corresponding time intervals served as controls to normalize the expression patterns. Tissue samples were collected as three biological replicates after each time interval.

### Biotic Stress Treatment

To check the expression pattern of rice ribosomal genes in response to biotic stress, we used the bacterial pathogen *Xanthomonas oryzae* pv. o*ryzae* that causes Bacterial Leaf Blight (BLB) of rice, which is one of the most severe yield constraints of rice worldwide (Sundaram et al., 2014). At the seedling stage, the infected leaves start to roll-up, and as the disease progresses, the leaves turn yellow and wilt, leading to drying up and death. This drastically reduces the total seed yield of the plant. The yield loss may be as high as 70% when plants are grown in conditions favorable to the disease (Ryan et al., 2011). The bacterial suspension of *Xanthomonas oryzae* pv. o*ryzae* was applied on the leaves of 2-month-old plants grown in greenhouse conditions, and leaf samples were collected after 11 days of infection. Leaf samples of untreated plants grown under similar conditions were used as a control to normalize the expression.

Because the transcript level of RPL10 was significantly up-regulated 11 days after treatment, we selected this gene in particular to analyze its expression at progressive time-points such as 3 h, 6 h, 1 day, 2 days, 3 days, up to 11 days post-infection of rice leaves with *Xanthomonas oryzae* pv. o*ryzae* pathogen. The qRT-PCR was performed with *Xanthomonas oryzae* pv. o*ryzae* treated and untreated samples collected as three biological and three technical replicates. Rice specific *act1* and β-*tub* genes were used as controls for normalization and the mean of the fold change was represented as bar diagrams constructed using SigmaPlot v11.

### RNA Isolation, cDNA Synthesis, and Quantitative-PCR (qRT-PCR)

Total RNA was isolated from stress-treated and untreated tissues using TriReagent (Takara Bio, UK) following the manufacturer’s protocol. The quality of extracted RNA was checked on 1.2% agarose gel prepared in TBE (Tris-borate-EDTA) buffer and quantified using Nanodrop. Total RNA (2 μg) was used to synthesize the first strand cDNA using reverse transcriptase (Takara Bio, UK). The cDNA was diluted in 1:7 proportions and 2 μl of it was used in qRT-PCR. Primers specific for each RPL gene sequence retrieved from RGAP-DB was designed using the primer-3^16^ online tool and 10 μM of each was used per reaction. The reaction conditions for qRT-PCR included an initial denaturation at 94°C for 2 min followed by 40 cycles of 94°C for 30 s, an appropriate annealing temperature for 25 s and an extension of 72°C for 30 s. At the end of the reaction, a melting curve step was inserted to analyze the specificity of amplification of each gene. Rice specific actin (*act1*) and tubulin (β-*tub*) were used as internal reference genes to normalize the expression patterns. The mean values of relative fold change, which was calculated as per ΔΔ*C*_T_ method (Livak and Schmittgen, 2001) obtained from each reference gene was considered as the final fold change in the transcript levels. Each qRT-PCR reaction was performed as three biological and three technical replicates.

The relative fold change of the 34 genes in 13 tissues and under five abiotic treatments was illustrated in the form of heat maps. A dendrogram was constructed to represent the Hierarchical clustering of relative fold change of 34 genes under each treatment using the GENE-E^17^ program.

## Results

### Genome-Wide Identification and Chromosomal Distribution of RPL Genes

To explore the cytoplasmic large subunit (60S) ribosomal gene family members in rice, we used a keyword search “ribosomal” in the putative function search of RGAP-DB and Phytozome databases, which resulted in the identification of 428 and 754 genes, respectively, and these included genes belonging to cytoplasmic 60S and 40S subunits and 50S and 30S subunits of chloroplast and mitochondrial ribosomes. Keyword search and homology-based identification through HMM are widely used practices in identifying genome-wide copies of the annotated genes (Kapoor et al., 2008; Liang et al., 2016). We then searched for genes starting with the prefix ‘L’ to select large subunit genes. This process excluded small subunit genes and identified 215 genes that included large subunit members of cytoplasmic (60S) and chloroplast and mitochondrial (50S) ribosomal subunits. We then shortlisted the cytoplasmic 60S subunit genes by their putative cellular localization using the information available in RGAP-DB. A similar process was applied in shortlisting the 60S subunit genes from Phytozome. Both these approaches identified 123 genes belonging to the cytoplasmic 60S subunit. Each of these genes was then confirmed by a BLAST search of their nucleotide and predicted amino acid sequences in other rice databases like RAP-DB^18^ and OryGenesDB. BLASTn and BLASTp results in NCBI and HMM of Pfam and NCBI conserved domain databases, respectively, further confirmed that these genes belong to the 60S ribosomal family by the presence of ribosomal domains.

**FIGURE 1:**
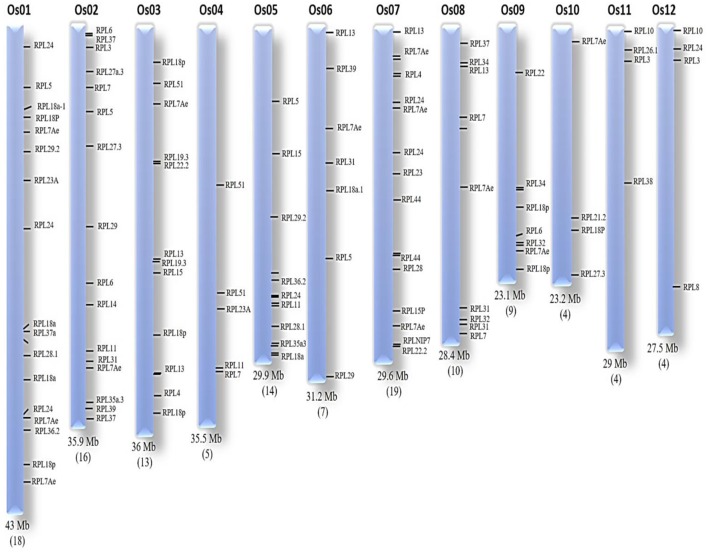
**Chromosomal organization of RPL genes.** The chromosomal number and size is represented at the top and bottom of each chromosome, respectively. The number of RPL genes is given within brackets at the bottom of each corresponding chromosome.

The locus numbers of 123 genes were submitted in OryGenesDB and, based on the output generated, the location of each gene on the corresponding chromosome was mapped manually using OryGenesDB. The location of these 123 genes was found on all chromosomes, indicating their wide distribution throughout the rice genome. Chromosome-7 has 19; chromosome-1, being the largest of rice chromosomes has 18; chromosome-2 has 16; chromosome-5 has 14; and chromosome-3 showed 13 genes. Chromosomes-9, 10, and 11 exhibited four genes each, while chromosomes-4, 8, and 9 evidenced 5, 10, and 9 RPL genes, respectively (**Figure 1**). The nucleotide sequence alignment of genes within an orthologous group exhibited 100% similarity, but their chromosomal locations are different. We selected the 34 candidate genes, each representing one orthologous group for a detailed characterization to understand their gene and protein structures, and comprehensive expression analysis in response to a wide range of stress treatments.

### Analyses of RPL Gene Structures

A comparative study between 34 RPL genes was performed to determine the number and position of introns and exons, GC-content and 5′ and 3′ untranslated regions. The number of introns varied from none to seven. The RPL10 and RPL18p have the highest number of introns (7), whereas RPL4, 8, 21.2, 23A, 26.1, 28, and 31 have only one intron. RPL7, 11, 24b, 27.3, and 38.2 do not contain any introns in their coding regions; these genes also have high GC-content. For example, RPL24b, 27.3 and 38 have 54, 60, and 51% GC-content, respectively. Genes with introns are reported to be crucial for gene expression, and a high number of introns is linked with increased expression of a gene (Callis et al., 1987; Karve et al., 2011). We observed that RPL18p and RPL10 with seven introns exhibited high expression in various tissues studied compared with other RPL members. Introns when particularly present at the start site or 5′ end of a gene have an enhanced ability for expression (Callis et al., 1987; Donath et al., 1995). RPL35a.3, 51, 32, 30e, 22, 19.3, 18a, 10, and 5 have their first introns within 500 bp regions from the transcription start site, which might be the reason for their constitutive expression in almost all the tissues studied. Similarly, the number of exons also varied among 34 RPL genes. Genes like RPL10 and RPL18p have the highest number of exons (8), whereas RPL7, 11, 24b, 27.3, and 38.2 have only one exon. Some of these RPL genes (RPL4, 13a, 14, 15, 23A, 28, 30e, 32, 35a, and 51) undergo alternative splicing to produce 2–5 splice-variants (**Table 1**) with similar nucleotide sequence. For expression analysis, we used the sequence of only one variant of a gene as it is difficult to design the primers for alternative splice forms exhibiting high nucleotide similarity. In addition, the 5′ and 3′ UTRs of these genes varied in size and positions. RPL28 has an unusually long UTR of 2.5 kb at the 3′ end (**Supplementary Figure S1**). **Table 1** provides detailed information about their gene structure, site of location in the genome and copy number of individual genes.

**Table 1:** Details of rice RPL genes.

Gene Id	Chromosome number	Location (bp)	Protein name	Gene Structure						Gene copies
					
				Gene Size (bp)	GC (%)	Introns	Exons	Alternative splice forms	Orientation	
LOC_Os11g06750	Os11	3294148–3297139	RPL3	2991	46%	5	5		5′–3′	2
LOC_Os03g58204.1	Os03	33143597–33146052	RPL4	2456	41%	2	2	2	3′–5′	3
LOC_Os05g11710.1	Os05	6649548–6651919	RPL5	2372	43%	4	4		5′–3′	5
LOC_Os04g39700.1	Os04	23660833–23663041	RPL6	2209	44%	3	4		3′–5′	2
LOC_Os02g10540.1	Os02	5550032–5552116	RPL7/L12	2085	46%		1		3′–5′	5
LOC_Os12g38000.1	Os12	23349992–23351879	RPL8	1888	49%	1	2		5′–3′	1
LOC_Os11g01420.1	Os11	252426–254919	RPL10	2494	41%	7	8		3′–5′	2
LOC_Os04g50990.1	Os04	30180315–30182052	RPL11	1738	44%		1		5′–3′	2
LOC_Os07g01870.1	Os07	523361–525795	RPL13a	2435	47%	2	3	2	3′–5′	4
LOC_Os03g37970.1	Os03	21086229–21088565	RPL13b	2337	46%	4	4		3′–5′	3
LOC_Os02g40880.1	Os02	24777727–24779637	RPL14	1911	42%	4	4	2	3′–5′	2
LOC_Os03g40180.1	Os03	22336536–22338324	RPL15	1789	48%	3	3	2	3′–5′	4
LOC_Os05g49030.1	Os05	28118285–28120495	RPL18a	2211	43%	3	3		5′–3′	3
LOC_Os01g67134.1	Os01	38973740–38976158	RPL18p/L5e	2419	43%	7	8		3′–5′	2
LOC_Os03g21940.1	Os03	12542185–12544692	RPL19.3	2508	43%	4	5		3′–5′	3
LOC_Os10g32820.1	Os10	17184530–17185759	RPL21.2	1230	46%	1	2		3′–5′	2
LOC_Os09g08430.1	Os09	4390880–4393872	RPL22	2993	41%	6	6		3′–5′	2
LOC_Os01g24690.1	Os01	13896291–13898264	L23A	1974	41%	2	2	3	5′–3′	2
LOC_Os01g59990.1	Os01	34682518–34685485	RPL24a	2968	42%	5	5		5′–3′	4
LOC_Os01g04730.1	Os01	2137763–2138996	RPL24b	1234	54%		1		5′–3′	3
LOC_Os11g05370.1	Os11	2382184–2383220	RPL26.1	1037	52%	1	2		3′–5′	3
LOC_Os02g18380.1	Os02	10700750–10701516	RPL27.3	767	60%	–	1		3′–5′	2
LOC_Os07g36090.3	Os07	21574435–21579332	RPL28	4898	42%	3	2	3	3′–5′	1
LOC_Os06g51530.1	Os06	31223501–31226586	RPL29	3086	46%	7	4		3′–5′	7
LOC_Os07g44230.1	Os07	26432701–26435257	RPL30e	2557	42%	6	6	2	3′–5′	1
LOC_Os06g21480.1	Os06	12412525–12415341	RPL31	2817	43%	1	2		5′–3′	4
LOC_Os09g32532.1	Os09	19418910–19421084	RPL32	2175	42%	3	3	2	3′–5′	4
LOC_Os09g24690.1	Os09	14691168–14693121	RPL34	1954	41%	3	4		3′–5′	3
LOC_Os05g48310.1	Os05	27695307–27697523	RPL35a.3	2217	46%	3	3	2	5′–3′	3
LOC_Os01g62350.1	Os01	36083051–36085662	RPL36.2	2612	38%	3	3		3′–5′	2
LOC_Os02g56990.1	Os02	34918111–34920148	RPL37	2038	44%	2	3		5′–3′	3
LOC_Os11g24610.1	Os11	14044207–14045356	RPL38	1150	516%		1		5′–3′	4
LOC_Os07g33898.1	Os07	20273588–20275840	RPL44	2253	42%	3	3		5′–3′	11
LOC_Os03g10930.2	Os03	5613250–5615444	RPL51	2229	43%	3	3	2	3′–5′	1

*RPL gene sequences were retrieved from RGAP-DB. Gene properties such as size, GC content, orientation in the genome, chromosomal distribution, copy number of each gene, number of introns and exons were studied in the OryGenesDB.*

### Domain Recognition, Ligand Binding Sites, and Phylogeny of Rice RPL Proteins

Among the 34 proteins, RPL4 is the largest with a predicted molecular mass of 44.5 kDa. All the RPL proteins have similar isoelectric points ranging from 9.5 to 12. The maintenance of similar *p*I values in RPL proteins might be to reduce coulombic repulsions as these proteins are interactive in nature. They have a varied percentage of α, β, and distorted regions with RPL proteins interacting with other r– and non-r proteins exhibiting high content of α-helices. For example, RPL7, which interacts with elongation factors-Tu and -G, has 55% α-helical structure. RPL13a, existing at the interface of RPL3 has 57% of α-helix content, whereas RPL29 that interacts with RPL23A and initiation factors has 48% α-helix content. Furthermore, all the RPL proteins have a GRAVY value < 0, which indicate their high hydrophilicity. The proteins with high hydrophilic nature tend to undergo conformational changes and form flexible structures with other molecules and also contribute toward inducing tolerance during stress conditions (Fuxreiter et al., 2004; Liang et al., 2016).

All the RPs are characterized by the presence of ribosomal domain(s). They also have several other domains that participate in interaction with other proteins. RPL 14, 19.3, 21.2, 24b, 26, and 27 have KOW-SH3 motifs at their N-terminal regions, which are involved in protein–protein interactions. KOW-motifs link RPs with transcription factors (Kyrpides et al., 1996). RPL18p (135–226 amino acids) has an FCD domain (FadR C-terminal Domain) that is involved in the regulation of transcription of genes. RPL18p also has a XPGN domain (208–281 amino acids) that is associated with cancer and Xeroderma pigmentosum in humans. RPL21.2 has cheY motif at the C-terminus activated by phosphorylation through histidine kinases. RPL22 has a DUF1087 domain (amino acids 1–67) that is involved in chromatin remodeling and a WWE domain that is associated with poly-ADP-ribosylation and ubiquitin-mediated proteolysis. RPL29 has a carboxyl-terminal domain (CTD) and proteins with such domains are related to pre-mRNA processing by binding with mRNA capping enzymes (Schwer et al., 2009). It also has RQC, a DNA binding domain found in RecQ helicases.

Ligand-mediated signal transmission is essential for proper functioning of a majority of proteins including certain RPs. However, recognition of the core structures or amino acids involved in protein–ligand interactions is of paramount importance for understanding the dynamic and kinetic properties of the proteins. Analysis of RPL proteins for the presence of sites for ligand binding reveals that out of 34, 20 RPL proteins have sites for binding with ligands (that include metals ions and cofactors) whereas no such ligand binding sites were observed in 14 proteins (RPL3, 4, 6, 10, 11, 13b, 14, 15, 18a, 24a, 29, 34, 36, and 37). RPL8 (Lys198), 13a (Pro114, 115), 19.3 (Glu179, Arg180), 21.2 (Arg70), 22 (Ile52), 24b (Leu118, Lys121, Ala122), 26 (Leu105), 28 (Tyr49), 38 (Lys35), and 44 (Gly51) have Mg^+2^ ion binding sites. RPL7, 31, and 51 bind with Cu^+2^ and RPL5 (Asp30, Thr33) and 23A (Lys104) have sites for binding with Ca^2+^ ions. RPL19, 35, 38, and 51 also have Zn^2+^ binding sites. RPL29 present at the interface of RPL23A binds with the cofactor FAD. The RPL35 binds with cofactors FUC and FAD. Metal ions in RPs serve important biological functions by interacting with nucleic acids, particularly RNA. Non-metal ligands are cofactors involved in catalytic activities. Cofactors in RPs ensure that the processes of protein initiation, amino acid assembly and termination are correctly undertaken (Maguire and Zimmermann, 2001). Because of this, RPs might have binding sites for both metal and non-metal ligands. The details of the protein properties such as size, *p*I, the percentage of α-helices and β-sheets, GRAVY indices, the presence of metal/non-metal ligands and their binding sites are detailed in **Supplementary Table S1**. The secondary structures of selected RPL proteins with ligand binding properties are represented in **Supplementary Figure S2**.

To evaluate the evolutionary relationships within the RPL protein family of rice, three phylogenetic trees were constructed using full-length amino acid sequences and amino acid sequences derived from ribosomal domains and Low Complexity Regions (LCR; **Supplementary Figure S3**). The unrooted phylogenetic tree was constructed by using a ‘neighbor joining algorithm’ with a bootstrap value of 1000. The homologous proteins having significant bootstrap value (>95%) were considered as having the highest similarity with respect to others.

The phylogenetic tree of full-length RPL proteins was divided into four clades or groups (A, B, C, and D). The RPL proteins, RPL24b and 26.1 have the highest similarity indicating that these two genes might have become duplicated recently in the rice genome. The phylogenetic relationship has also been used for gene function identification and the proteins with the highest similarity perhaps exhibit similar functions and expression patterns (Lijavetzky et al., 2013). The expression of RPL24b and RPL26.1 was similar in shoots indicating their similar roles in shoot growth and development; their expression was also similar in oxidative stress indicating functional similarity.

The phylogenetic trees of two separate domains, the ribosomal domain and LCR were also divided into groups to check domain-wise similarity. The ribosomal-domain analysis showed maximum sequence similarity between RPL24b and 26, which exhibited similarity with RPL18a and 19.3 proteins. RPL18a and 19.3 showed similar expression patterns in different tissues like spikes, endosperm, plumules, radicles, 45 days shoot and root tissues and root–shoot transition indicating their functional similarity during growth and development. All the RPL proteins except RPL10, 11, 14, 21.2, 35, 36, 37, 38, and 51 have predicted LCR. The phylogenetic analysis of LCR showed that RPL6 and RPL26 exhibited the maximum sequence similarity followed by RPL5 and 31 and RPL27 and RPL8, whereas RPL3 formed a separate clade, which showed its possible divergence from the other RPL proteins.

### *In silico* Analysis of Putative Promoter Regions of Rice RPL Genes

The expression studies showed that many RPL genes are differentially regulated in various tissues and under various abiotic treatments. To assess whether this differential regulation is due to the presence of stress or signal-responsive elements in their regulatory regions, nucleotide sequences ≤1 kb upstream to each of the 34 genes were retrieved and searched using the PlantCARE database. This analysis resulted in the identification of multiple stress-responsive elements in the putative promoter regions of all the genes. Abiotic stress-responsive elements that are associated with heat and cold temperatures such as HSE (Heat Stress Elements) and LTR (Low-Temperature Response) and dehydration stress such as MBS (Myb Binding Site) are widely distributed within the putative promoter regions of RPL genes. MBS is a binding site for MYB-related transcription factors that are involved in the regulation of genes responsive to water-deficit conditions (Urao et al., 1993). The presence of these elements in the promoter regions suggests that the corresponding genes become activated under water stress or drought conditions. In addition to abiotic stresses, elements that respond to phytohormones such as ABA (ABRE-Abscisic acid responsive element and Motif IIb), MeJa (TGACG-motif and CGTCA-motif), SA (TCA-motif), Gibberellic acid (GARE-Gibberellic acid responsive element), and Auxin (TGA-motif and AuxR-Auxin responsiveness) are also present in multiple copies.

**FIGURE 2:**
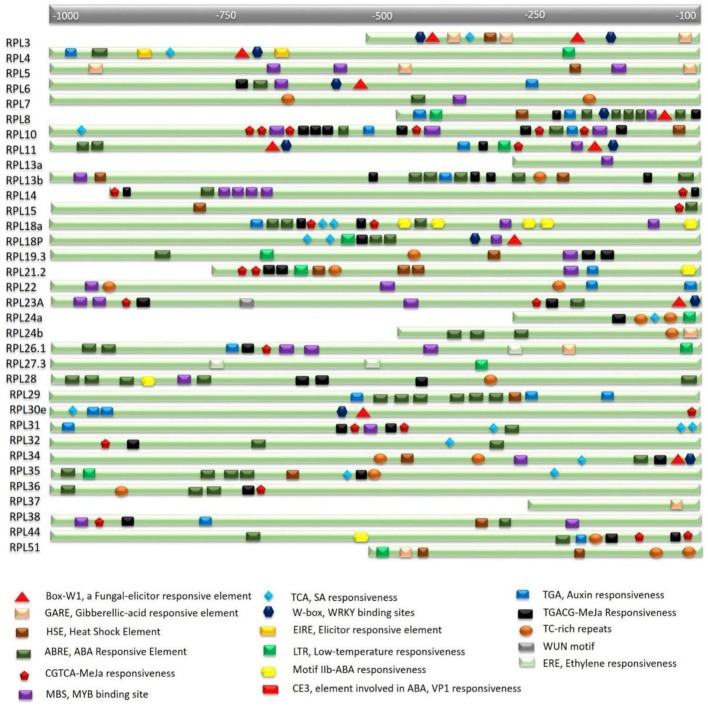
***In silico* analysis of rice RPL promoters for the identification of *cis*-regulatory elements.** Nucleotide sequence ≤1 kb upstream of the transcription start site of each gene carries multiple stress and signal-responsive elements. Each element is represented with a different shape and color which is described at the bottom of the figure. A scale at the top indicates the putative localization of corresponding elements.

Except RPL37, which did not exhibit any abiotic-responsive element in its upstream region, the putative promoters of all other genes had one or the other stress-responsive elements. MBS, ABRE, TGACG, and CGTCA motifs are commonly found in multiple copies. RPL8 has five repeats of ABRE and two repeats of each MBS, TGACG, CGTCA and TGA elements. RPL10 exhibited five repeats of each TGACG and CGTCA motifs that respond to MeJa treatment and three copies of MBS elements. RPL14 has four repeats of dehydration responsive elements. RPL18a showed four and three copies of Motif IIb and ABRE, respectively, that respond to ABA and two copies of TGACG and CGTCA motifs. RPL28 showed five repeats of ABRE and four repeats of MeJa responsive elements. RPL29 has six copies of ABRE and three copies of TGA element. RPL31 showed three copies of TCA element and two repeats of TGACG and CGTCA motifs. RPL35 has four copies of ABRE and two copies of TCA element. RPL36 and 38 had three copies of ABRE and MBS elements, respectively. RPL44 has two copies of ABRE, CGTCA and TGACG motifs (**Figure 2**). In addition, TC-rich repeats that are involved in defense and stress-responsiveness (Diaz-De-Leon et al., 1993), W-box motifs which are the binding sites for stress-responsive WRKY transcription factors (Eulgem and Somssich, 2007), a WUN-motif, a wound-responsive element that is associated with biotic stress (Jiang et al., 2014), a Box-W1 motif, a fungal elicitor element that binds with WRKY33 transcription factor in response to phytopathogens (Rushton et al., 1996; Lippok et al., 2007) are present in single copies in the putative *cis*-elements. **Supplementary Table S2** presents a detailed analysis of both abiotic and biotic responsive elements and their repeats in the putative promoter regions.

### Spatial Expression of Rice RPL Genes

To obtain insights into the tissue-specific and native expression patterns of RPL genes, we studied the expression of 34 RPL genes in 13 different tissues including 16 h embryo and endosperm, plumule and radicle of 3-day-old seedlings, root and shoot tissues of 7-day-old seedlings, 2-month-old tissues of mature flag leaf, shoot, root, root–shoot transition, partially filled grains, flowers and spikes. The primer details of 34 RPL genes used in the expression analysis were provided in **Supplementary Table S3**.

**FIGURE 3:**
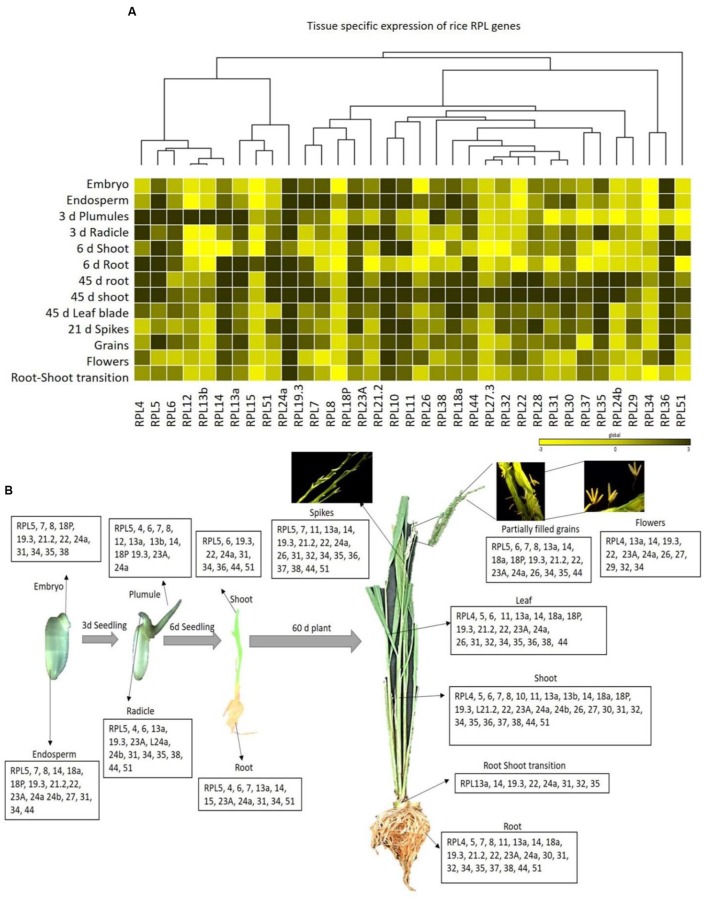
**Tissue-specific expression of rice RPL genes.** **(A)** The qRT-PCR of 34 RPL genes was performed in 13 different tissues and the level of expression was normalized with rice actin. The mean values of fold change of biological and technical triplicates were represented in the form of heat maps. A dendrogram was constructed to represent the Hierarchical clustering. **(B)** The RPL transcripts that are significantly up-regulated in each tissue are represented pictorially at the bottom.

Two-month-old shoot and root tissues induced an up-regulation of 30 and 22 RPL genes, respectively, which is larger than any other tissue. Out of 34, root–shoot transition and grains induced the expression of 17 RPL genes. Embryo, 6 days root and floral organs induced the expression of 12 RPL genes, whereas endosperm, plumule, radicle, 6 days shoot, flag leaf, and spikes induced the expression of a total of 16, 13, 14, 10, 21, and 19 RPL genes, respectively. RPL5 and RPL24a were highly up-regulated in all the tissues. The expression of RPL27 and RPL37 was detected only in three tissues; endosperm, 45 days shoot, flowers and 6 days shoot, 45 days root and flowers, respectively. RPL8 and RPL15 were expressive only in endosperm and 6 days roots, respectively, but non-expressive in the remaining tissues studied. Ten RPL genes *viz*., RPL5, 7, 8, 18P, 19.3, 21.2, 22, 24a, 31, and 34 were commonly up-regulated in embryo and endosperm indicating that they can be implicated in early embryonic development. RPL4-6, 13a, 19.3, 23A, and 24a were up-regulated in plumules and radicle suggesting their role in root and shoot initiation. RPL5, 6, 24a, 31, 34, and 51 were highly expressive in shoot and root tissues of 7-day-old seedlings. RPL4-6, 11, 13a, 14, 18, 19, 21.2, 22, 23A, 24a, 31, 34, 35, 38, and 44 were commonly up-regulated in root, shoot and flag leaf indicating that these genes are involved in vegetative growth and plant maturity. RPL13a, 14, 19.3, 22, 24a, 26, and 34 were highly expressive in spikes, flowers and partially filled grains indicating that these are likely associated with the development of reproductive organs and grain filling. RPL13a, 14 and 24a were expressive from the 6-day-old seedling stage to the grain filling stage in all the tissues studied indicating that they play a major role in the growth and development of both vegetative and reproductive organs such as root, shoot, flowers and grains. RPL5, 19.3, 23A, and 24a were expressive in mitotically active tissues like embryo, endosperm, plumule, and radicles suggesting that these genes are involved in the early maturity and emergence of shoot and root. RPL10 and RPL29 were specifically expressed only in endosperm and flowers, respectively (**Supplementary Table S4**). The spatial expression of 34 RPL genes in 13 different tissues has been represented in the form of heat maps generated by incorporating the qRT-PCR data obtained from tissue samples collected as three biological replicates (**Figure 3**).

### Differential Transcriptional Regulation of RPL Genes under Various Abiotic Stress Treatments

The motivation for studying rice large subunit ribosomal genes in stress-response stems from our results on activation-tagged mutant population generated for an important agronomical trait called water-use efficiency (Moin et al., 2016). The mutants with sustained/improved seed productivity under the conditions of limited water availability were selected for flanking sequence analysis and subsequently for studying the expression pattern of the enhancer tagged genes. The seven short-listed mutant plants that appeared to have high productivity were then characterized with physiological parameters related to WUE such as measuring their photosynthetic efficiency and carbon isotope analysis. Among these, two mutants were found to have ribosomal large subunit genes, RPL6 and RPL23A activated by the integrated enhancers. The presence of multiple stress-responsive elements in their putative promoter regions and their significant up-regulation in response to ABA, NaCl, and dehydration stresses further corroborated our findings (Moin et al., 2016). This study not only suggested that RPL6 and RPL23A are potential candidates for abiotic-stress amelioration, but more importantly it provides a basis for the exploration of other members of large subunit ribosomal genes for stress-responsiveness.

Taking a cue from these observations, we assessed the abiotic stress responsive roles of other rice 60S ribosomal genes. For this, we selected 34 genes, one from each orthologous group as described earlier and comprehensively studied the differential transcriptional regulation of 34 genes under phytohormone (MeJa and SA), temperature (heat; 42°C and cold; 4°C) and oxidative stress treatments in shoot and root tissues at six different time intervals (5 min, 3 h, 6 h, 12 h, 24 h, and 60 h). After applying the abiotic treatments, tissue samples were collected as early as 5 min to check the immediate responsiveness of the RPL genes and continued up to 60 h. All the RPL genes responded to the treatments in the form of either up or down-regulation.

The genes that exhibited ≥3-fold transcript level on the log_2_ scale were considered as significantly up-regulated. MeJa, SA, and cold treatments induced the up-regulation of more genes (>60%) than heat and H_2_O_2_ treatments, which caused the down-regulation of 75% of the genes. In shoots, MeJa and SA-induced the up-regulation of 27 (79%) RPL genes each, cold treatment up-regulated 19 genes (55%), while heat and H_2_O_2_ treatments up-regulated 6 (17%) RPL genes each. In roots, MeJa, SA, cold, heat, and H_2_O_2_ treatments up-regulated 19 (55%), 22 (64%), 16 (47%), 6 (17%), and 14 (41%) RPL genes, respectively. Genes that were up-regulated in both the shoot and root tissues include; RPL7, 8, 12, 13b, 18P, 19.3, 24a, 32, 35, and 51 under MeJa treatment, whereas SA-induced the expression of RPL7, 8, 12, 13b, 19.3, 24a, 26, 32, 35, and 51. Genes such as RPL6, 7, 23A, 28, 32, 35, and 37 were up-regulated in cold treatment, while RPL6, 12, 23A, and RPL18a and 13a were up-regulated under heat and H_2_O_2_ treatments, respectively.

To study the detailed regulation of RPL genes at various time points, we categorized the genes that responded within 5 min to 3 h after treatment as immediate-early (IE), those that responded between 3 h to 12 h as early (E) and those that were regulated after 12 h of treatment up to 60 h were considered as late (L) responding genes. The majority of the genes that were up-regulated had responded immediately within 5 min to 3 h after the onset of the stress. In shoots, among the genes that were expressive, a total of 17, 21, 19, 6, and 1 genes belonged to the IE-responsive class with instantaneous up-regulation under MeJa, SA, cold, heat, and H_2_O_2_ treatments, respectively. Among this IE-responsive class of genes, some continued to maintain a high level of expression at all the time points observed, while others exhibited a split in the expression followed by again an increase in the level of their expression. These probably form an important set of genes that respond to environmental stresses and might function as an immediate defense after the onset of the stress (Kawasaki et al., 2001).

The other class of IE genes was down-regulated after an IE response. Under MeJa and SA treatments, RPL7, 8, 12, 13b, 19.3, 24a, 28, and 35 maintained a high level of expression throughout the duration of stress in both shoot and root tissues. However, the level of transcriptional up-regulation varied with some exhibiting a very high level of up-regulation up to 100-fold (RPL8, 12, 19.3, 24a, and 35), while some had moderate expression up to 30-fold (RPL7) and others showed low transcript levels with <10-fold (RPL28). RPL6, 7, 12, and 24a exhibited a consistent up-regulation under cold and heat treatments, of which RPL6 and 12 became up-regulated more than 50-fold. In H_2_O_2_ treatment, RPL18a was up-regulated in both roots and shoots up to 65-fold whereas 24a, 24b, 30, and 34 showed significant up-regulation in roots. Since stress signals are transmitted through the roots to other parts of the plant body, genes that were significantly up-regulated particularly in roots might have an important role in combating the stress and providing early defense.

**FIGURE 4:**
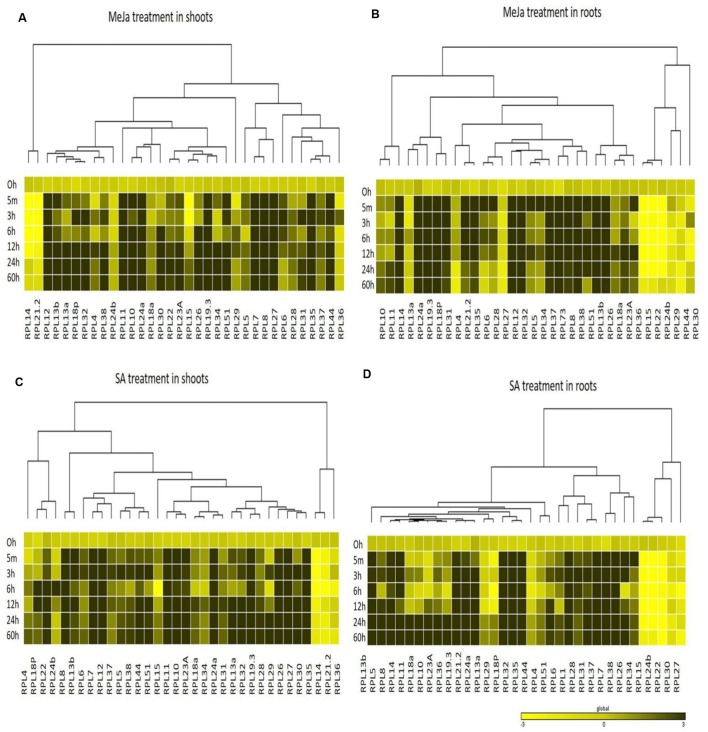
**Heat map representation of RPL genes in rice treated with MeJa and SA.** Seven-day-old rice seedlings were exposed to different abiotic stresses such as MeJa; 100 μM **(A,B)** and SA; 3 mM **(C,D)** at six different time points as indicated on the left. The qRT-PCR is used to determine the expression levels of RPL genes and the fold change was normalized using ΔΔ*C*_T_ method relative to that in unstressed seedlings dipped in water at corresponding time points. Rice actin (*act1*) and β-*tub* genes were used as internal controls. Three biological replicates and two technical replicates were included in the study. A dendrogram was constructed to represent the Hierarchical clustering of genes.

**FIGURE 5:**
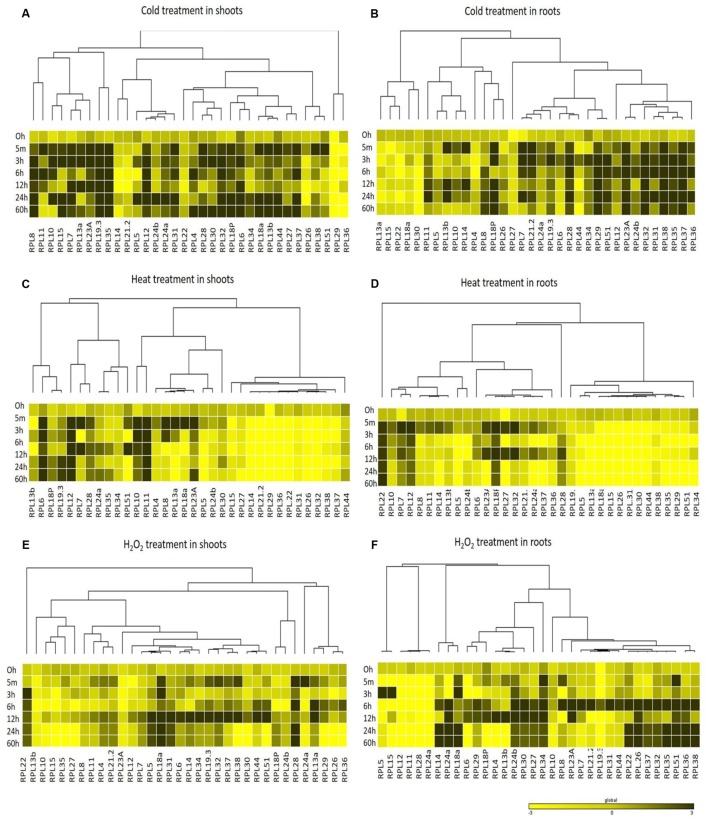
**Heat map representation of RPL genes in rice in response to cold, heat and H_2_O_2_ treatments.** Seven-day-old rice seedlings were exposed to different abiotic stresses such as cold stress at 4°C **(A,B)**, heat stress at 42°C **(C,D)** and oxidative stress with H_2_O_2_; 10 μM **(E,F)** at six different time points as indicated on the left. The qRT-PCR is used to determine the expression levels of RPL genes and the fold change was normalized relative to that in unstressed seedlings dipped in water at corresponding time points. Rice actin *(act1*) and β-tubulin were used as internal reference genes. Three biological replicates and two technical replicates were included in the study. A dendrogram was constructed to represent the Hierarchical clustering of genes.

**FIGURE 6:**
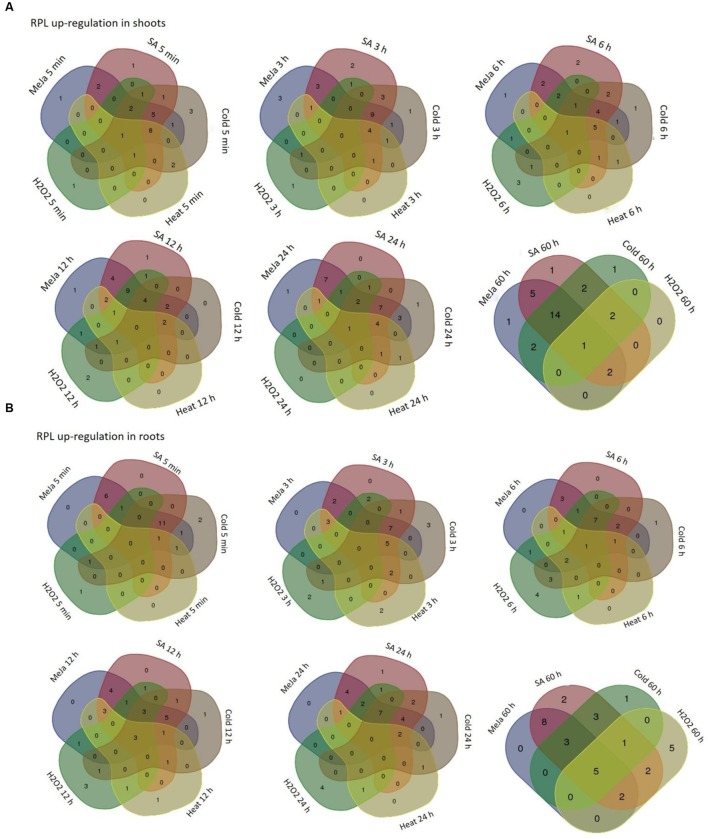
**Overlap in the up-regulation of rice RPL genes under five abiotic conditions.** The RPL genes that exhibited ≥3-fold transcript level on the log_2_ scale were considered as significantly up-regulated while others were considered as down-regulated or without any change in expression. Venn diagrams are used to show the overlap in the up-regulation in shoot **(A)** and root **(B)** tissues.

**FIGURE 7:**
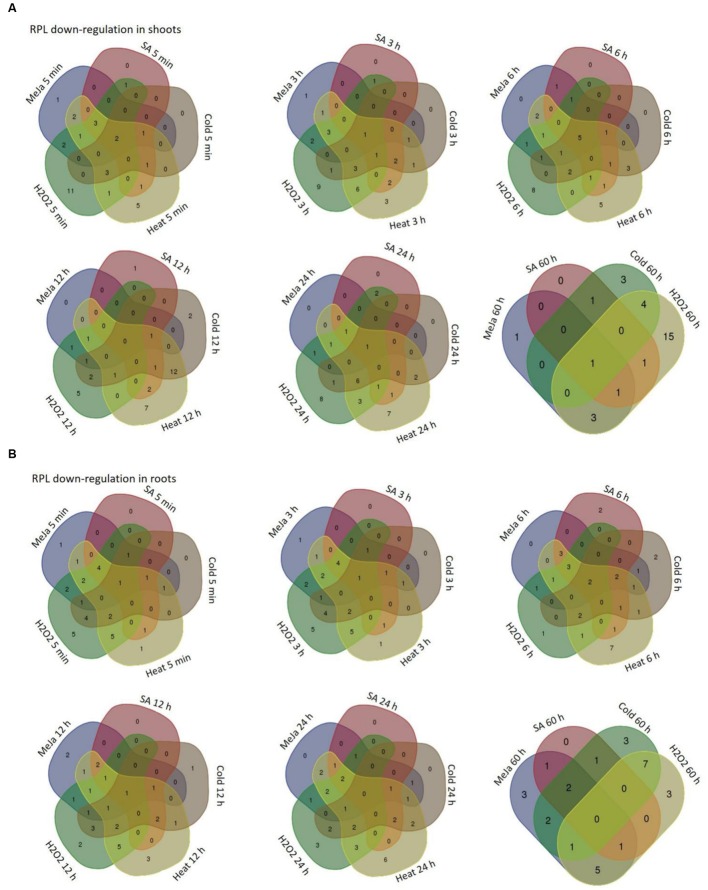
**Overlap in the down-regulation of rice RPL genes under five abiotic conditions.** The RPL genes that exhibited <3-fold transcript level on the log_2_ scale were considered as down-regulated with respect to others. Venn diagrams are used to show the overlap in the down-regulation in shoot **(A)** and root **(B)** tissues separately.

The differential expression patterns in response to MeJa and SA (**Figure 4**) and cold, heat and oxidative treatments (**Figure 5**) have been represented in the form of heat maps. These were generated by incorporating the mean values of fold change normalized using ΔΔ*C*_T_ method obtained from three biological and three technical replicates. The overlap in the up-regulation (**Figure 6**) and down-regulation (**Figure 7**) of 34 RPL genes in both shoot and root tissues were represented as Venn diagrams. **Supplementary Tables S5** and **S6** provide a detailed list of genes that exhibited overlap in the up and down-regulation, respectively, in shoot and root tissues at each time point.

### Differential Transcriptional Regulation of RPL Genes in Response to Treatment with the *Xanthomonas oryzae* pv. o*ryzae* Pathogen

**FIGURE 8:**
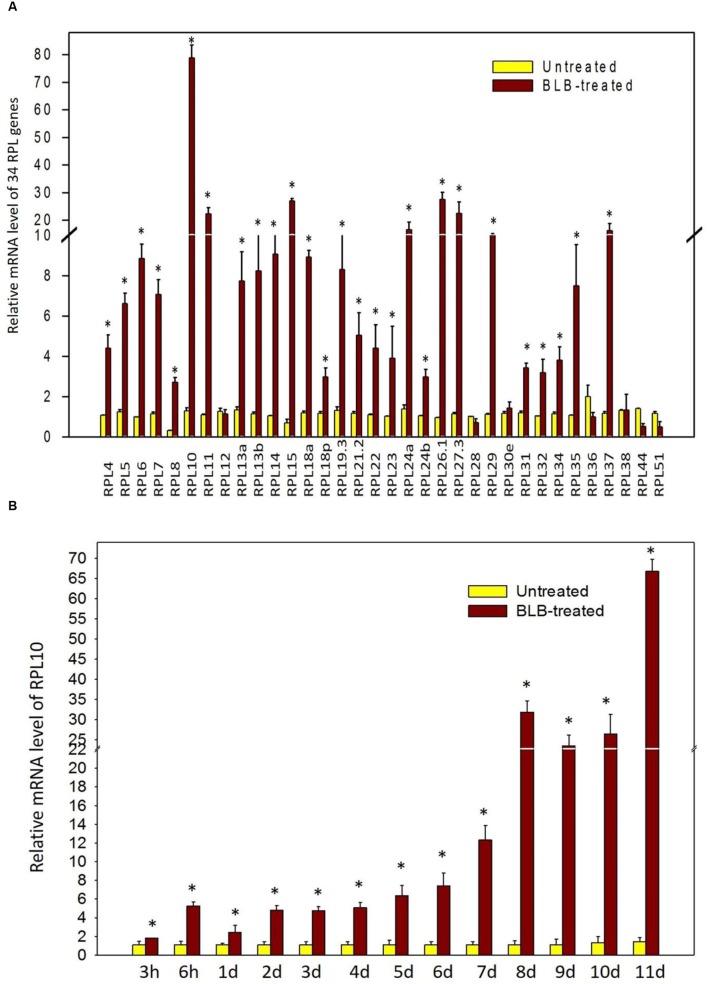
**Expression of RPL genes upon infection of rice with the *Xanthomonas oryzae* pv. o*ryzae* pathogen.** The expression of RPL genes was determined in response to the bacterium, *Xanthomonas oryzae*, which causes leaf blight. **(A)** The bacterial suspension was applied on 60-day-old rice plants, and after 11 days of treatment, leaf samples were analyzed for differential transcript levels of 34 RPL genes. **(B)** Since the up-regulation of RPL10 was significant, we analyzed its expression at progressive time courses such as 3 h, 6 h, 1 day, 2 days, 3 days, 4 days, 5 days, 6 days, 7 days, 8 days, 9 days, 10 days, and 11 days post-infection with *Xanthomonas oryzae* pv. o*ryzae* pathogen. The expression was normalized with untreated samples of same age grown under identical conditions. The statistical significance was calculated using one-way ANOVA at *P* < 0.05 and represented with asterisks.

The qRT-PCR analysis of 34 genes showed that they are differentially regulated under abiotic treatments, with many of them becoming significantly and immediately up-regulated. Hence, we simultaneously examined the expression levels of 34 RPL genes in response to the *Xanthomonas oryzae* pv. o*ryzae* pathogen that causes BLB of rice. Out of 34 genes, 6 (17%) were down-regulated, RPL38 was non-responsive, and the remaining genes became activated (80%). RPL12, 28, 30, 36, 44, and 51 were among those that were down-regulated, and the transcript level of RPL38 did not change significantly, while all other genes studied were up-regulated. Among those that were expressive, RPL10, 11, 15, 24a, 26, 27, and 37 up-regulated more than 10-fold. The transcript level of RPL10 was the highest with more than 75-fold up-regulation (**Figure 8A**). In addition to significant expression at 11 days, the transcript level of RPL10 also exhibited a gradual increase at 3 h, 6 h, 1 day, and 2 days up to 7 days post-infection with the *Xanthomonas oryzae* pv. o*ryzae* pathogen (**Figure 8B**).

## Discussion

Stress factors such as heat, cold, dehydration, and pathogen attack can exacerbate the global agriculture system with an estimated >50% crop yield loss per annum (Wang et al., 2003). Each of these stresses elicits a cascade of signaling pathways to ensure plant survival. It is important to identify the genes that contribute to sustainable plant productivity under conditions of stress to further improve their productivity potential. Although more than 500 genes have been overexpressed and characterized in rice for stress-tolerance, many remain to be identified and examined.

In the present work, we report on the comprehensive expression profiling of rice ribosomal large subunit genes under multiple abiotic and biotic stress treatments at progressive time points and also identified their putative promoter sequences. The information provided here can be exploited further in the functional characterization of these stress-responsive genes, which might help in augmenting rice yields by generating independent transgenic plants. We also identified the genes that exhibited an overlap in the expression patterns in response to two or more stresses. We propose that such genes are particularly promising in bringing about the tolerance to multiple stresses as the presence of a second stress factor can enhance the detrimental effects of the first one (Atkinson and Urwin, 2012).

Ribosomal genes encode proteins that are the components of the two-subunit ribosomal complex, which together with the members of the same group and other proteins participate in protein synthesis. There have been limited reports on the role of ribosomal genes in the stress-responses. We had generated an activation-tagged transgenic rice plant population carrying CaMV35S tetrameric enhancers. These rice mutants were screened for water-use efficiency by growing them under the provision of limited water supply compared to the level that required for normal growth of rice. Flanking sequence analysis of selected mutants having sustained growth and productivity under the condition of limited water availability revealed the activation of the two ribosomal genes (RPL6 and RPL23A) by the integrated enhancers (Moin et al., 2016). This has persuaded us further to analyze the importance of several of RPL genes in stress-responses. We therefore, performed a comprehensive native tissue-specific and differential expression of 34 RPL genes under various abiotic and biotic stress conditions at different time intervals.

The availability of full-length and high-quality rice genome sequence and databases, further helped us to exploit the information on these genes. Based on the information available in rice databases, we identified 123 genes that are the components of rice ribosomal large subunit, of which 2–3 genes exist as identical gene copies in the genome. These genes are distributed throughout the 12 chromosomes of rice genome with chromosome 7 and 1 having the highest number of genes. The present investigation that reports on the analysis of native and differential expression of the RPL gene family also corroborated the earlier reports that RPL genes are regulated spatio-temporally (Sormani et al., 2011; Carroll, 2013; Zheng et al., 2016). In rice, RPL genes appear to be developmentally regulated as they are widely expressed in all the 13 tissues studied starting from as early as embryonic initiation to plant maturity. Among the 19 RPL genes that were expressive in grain filling stage, the expression of RPL5, 7, 13a, 14, 19.3, 22, 24a, 34, and 35 were conspicuously detected. Further characterization of these genes would throw useful insights into their role in grain production, which is a significant yield-related trait in rice.

Although many of the RPL genes were expressive in all the tissues, they cannot be considered as house-keeping as their level of expression changed in response to environmental signals. Similar expression profiling of small and large subunit genes was reported in response to macro-elements deficiency in *Arabidopsis* in which about 244 among 249 RP genes became up-regulated (Wang et al., 2013). The up-regulation of the RPL genes is likely to maintain or improve protein synthesis and hence, proper functioning of ribosomes, the basic cellular moieties under the conditions of stress (Kim et al., 2004). Plants being sessile acclimate to environmental cues by undergoing many metabolic changes, one of them being increased protein turnover that includes both protein biosynthesis and ubiquitination (Kosová et al., 2014). Proteomic studies revealed variations in the levels of translation-related proteins such as initiation factors, elongation factors, and proteins of both small and large subunits during the process of acclimation particularly, to dehydration, salt and temperature stresses in cereals (Fatehi et al., 2012; Budak et al., 2013; Ghabooli et al., 2013; Gharechahi et al., 2014).

In addition to their significant up-regulation, the presence of multiple *cis*-regulatory elements in the putative promoter regions of RPL genes further corroborates our findings that these genes might also play a role in alleviating plant biotic and abiotic stress. RPL6 and RPL23A, in addition to their role in WUE, became up-regulated in almost all the stresses studied illustrating their possible involvement in inducing tolerance to abiotic stresses (Moin et al., 2016). Cold, MeJa, and SA treatments induced the up-regulation of a majority of RPL genes, while H_2_O_2_ and heat treatments down-regulated 75% of the genes.

The up-regulation of RPL genes by cold treatment is to enhance the process of polypeptide synthesis at low temperatures (Kim et al., 2004). RPL7, 8, 12, 13b, 19.3, 24a, 28, and 35 were up-regulated and constantly maintained a high level of expression throughout the duration of stress in response to SA and MeJa, the two phytohormones involved in plant defense against pathogen attack. These genes also contain TC-rich repeats, which are known for their involvement in plant defense and stress response (Diaz-De-Leon et al., 1993). RPL18a, 24a, 24b, 30, and 34 were expressed at higher levels when exposed to H_2_O_2_ treatment. This may reflect that these genes might have potential in combating oxidative stress. High temperature appeared to cause detrimental effects on the expression of RPL genes as heat stress had down-regulated >75% of the genes. RPL6, 12, and 23A were among those whose expression was detected. Down-regulation of RPL genes under high temperature might be because of decreased stability of RNA molecules with increasing temperatures.

Infection with *Xanthomonas oryzae* pv. o*ryzae*, which causes BLB also up-regulated a large number of RPL genes. Among those that were expressive (RPL10, 11, 15, 24a, 26, 27, and 37), the expression of RPL10 was more evident as its transcript levels gradually increased from 3 h post-infection and reached a peak at 11 days after treatment. Also, RPL10 was activated in shoots under MeJa and SA up to 60 h after treatment, further suggesting its involvement in biotic stress response.

Our exploration on the detailed expression analysis underpins that these genes regulate tissue-specific development and respond rapidly to the environmental cues and might function as facilitators of immediate defense against stresses. The coordinated transcriptional up-regulation of translation-related genes is a necessity for the cells to maintain the crucial functions of ribosomes under the conditions of stress. The increase in the expression of RPL genes under a wide range of stress-treatments including both biotic and abiotic conditions demonstrate that these are potential targets for the manipulation of stress-tolerance in rice and other related cereal crops as well. However, the level of tolerance induced by each of these genes needs to be analyzed by their independent overexpression in the transgenic rice plants.

## Author Contributions

PK and MM designed the experiments. MM performed all the experiments. AB, MD, and AS helped in the analysis of qRT-PCR data. SM performed the *Xanthomonas oryzae* pv. o*ryzae* infection on rice leaves. MM and PK prepared the manuscript.

## Conflict of Interest Statement

The authors declare that the research was conducted in the absence of any commercial or financial relationships that could be construed as a potential conflict of interest.

## Footnotes

1 http://rice.plantbiology.msu.edu/index.shtml

2 https://phytozome.jgi.doe.gov/pz/portal.html

3 http://blast.ncbi.nlm.nih.gov/Blast.cgi

4 http://pfam.xfam.org/

5 http://www.ncbi.nlm.nih.gov/Structure/cdd/wrpsb.cgi

6 http://orygenesdb.cirad.fr/tools.html

7 http://gsds.cbi.pku.edu.cn/

8 http://psort.hgc.jp/form.html

9 http://www.genome.jp/tools/clustalw/

10 http://www.megasoftware.net/

11 http://smart.embl-heidelberg.de/

12 http://web.expasy.org/cgi-bin/protparam/protparam

13 http://www.sbg.bio.ic.ac.uk/phyre2/html/page.cgi?id=index

14 http://www.sbg.bio.ic.ac.uk/3dligandsite/

15 http://bioinformatics.psb.ugent.be/webtools/plantcare/html/

16 http://bioinfo.ut.ee/primer3-0.4.0/

17 http://www.broadinstitute.org/cancer/software/GENE-E/

18 http://rapdb.dna.affrc.go.jp/

## Abbreviations

H_2_O_2_: hydrogen peroxide
MeJa: methyl jasmonate
RP: ribosomal protein
RPL: ribosomal protein large subunit
SA: salicylic acid
