# Supplementary material for: Rice Ribosomal Protein Large Subunit Genes and Their Spatio-temporal and Stress Regulation
Source: Front Plant Sci. 2016 Aug 24;7:1284. doi: 10.3389/fpls.2016.01284 (PMC4995216; doi:10.3389/fpls.2016.01284)
Supplement: Supplementary file 1 [file Data_Sheet_1.DOCX]

**Supplementary Figure 1**


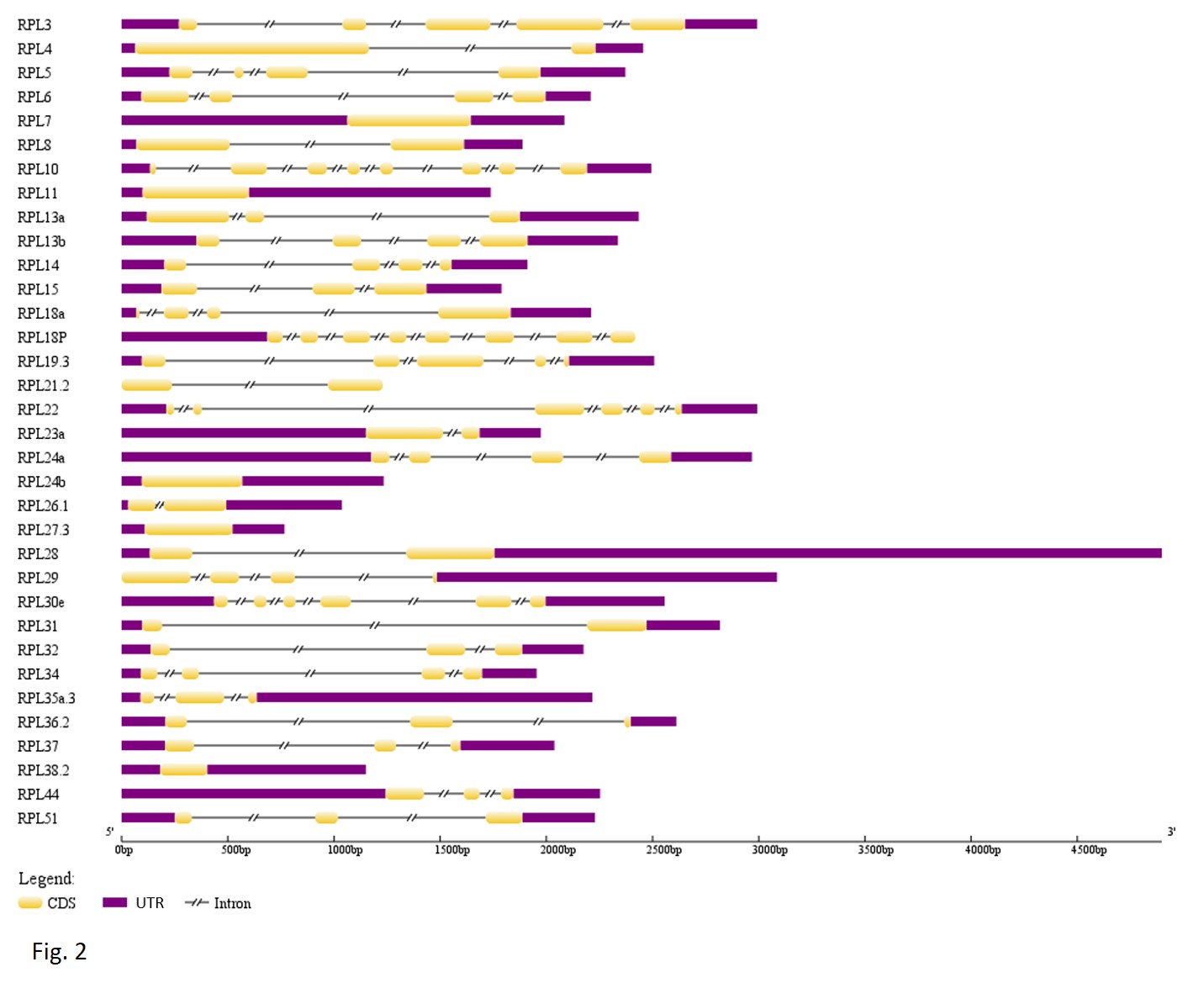


**Representation of structures of 34 RPL genes**

The yellow and blue boxes represent exon and UTR regions, respectively whereas introns are indicated as the split line.

**Supplementary Figure 2**

**
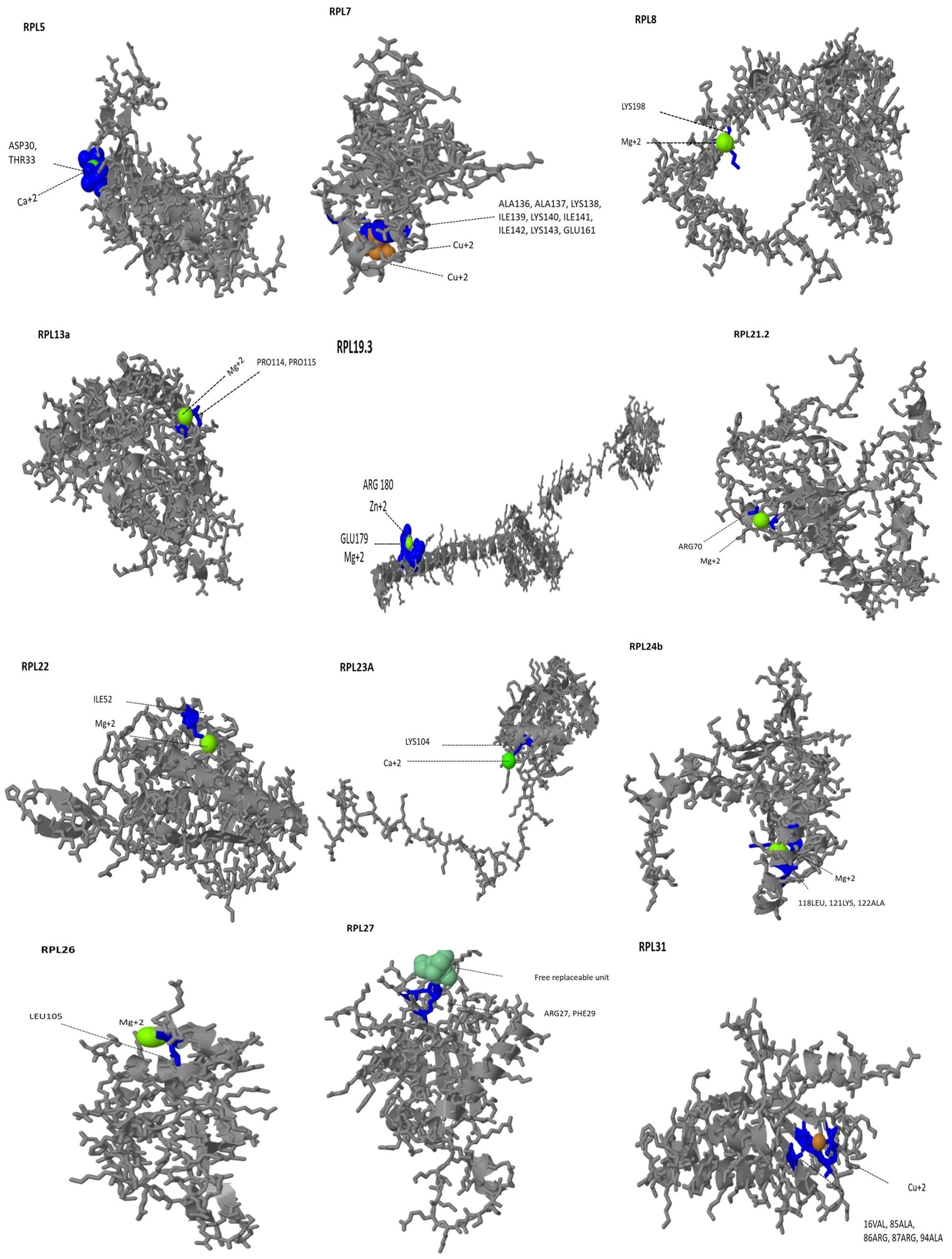
**

**
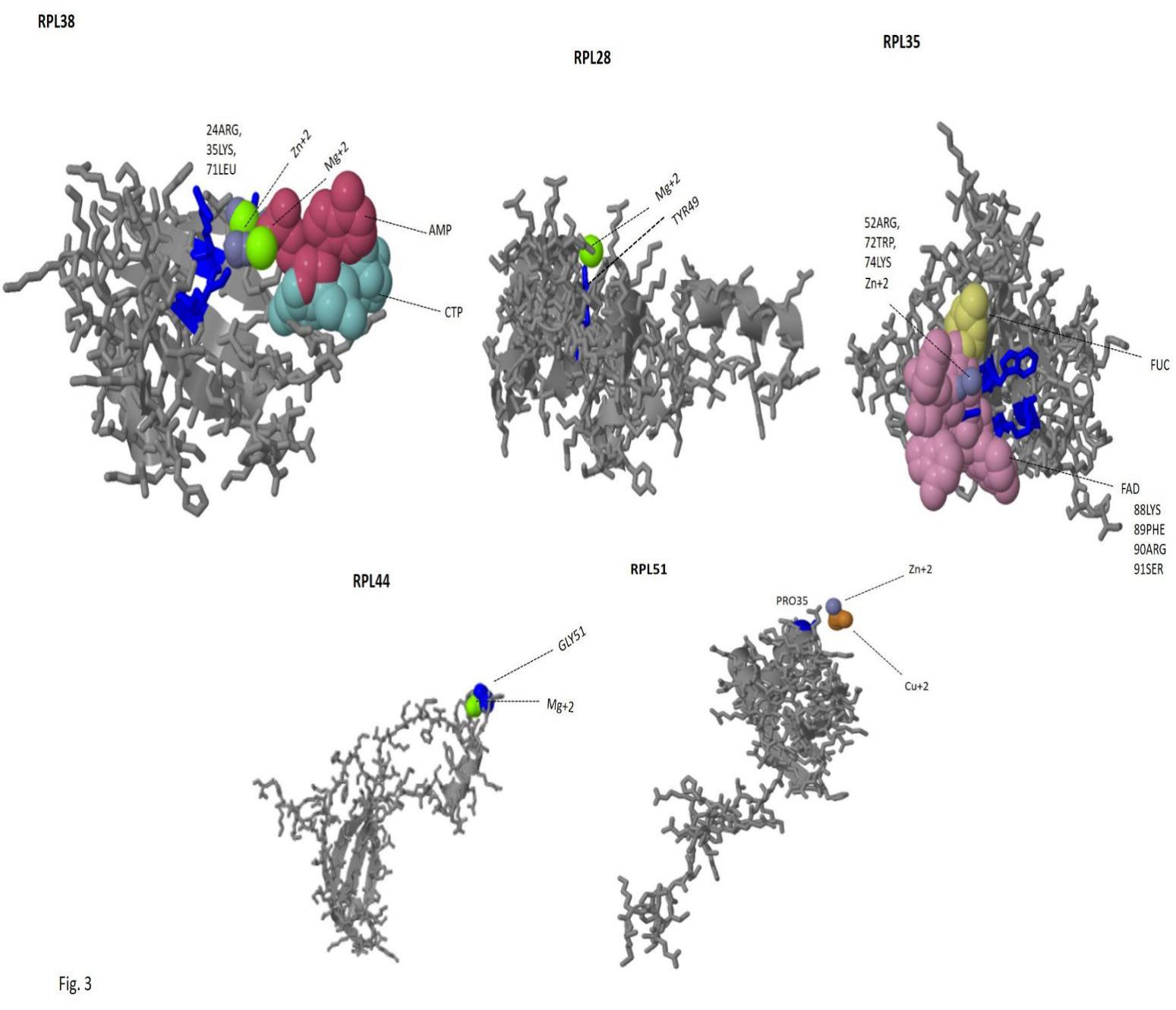
**

**RPL protein predicted secondary structures and ligand binding sites**

The secondary structures of selected 17 RPL proteins with predicted ligand binding sites. The metal ligands or cofactors are represented with different colored balls followed by amino acids involved in ligand binding indicated as split lines.

**Supplementary Figure 3: Phylogenetic relations within RPL proteins**

**
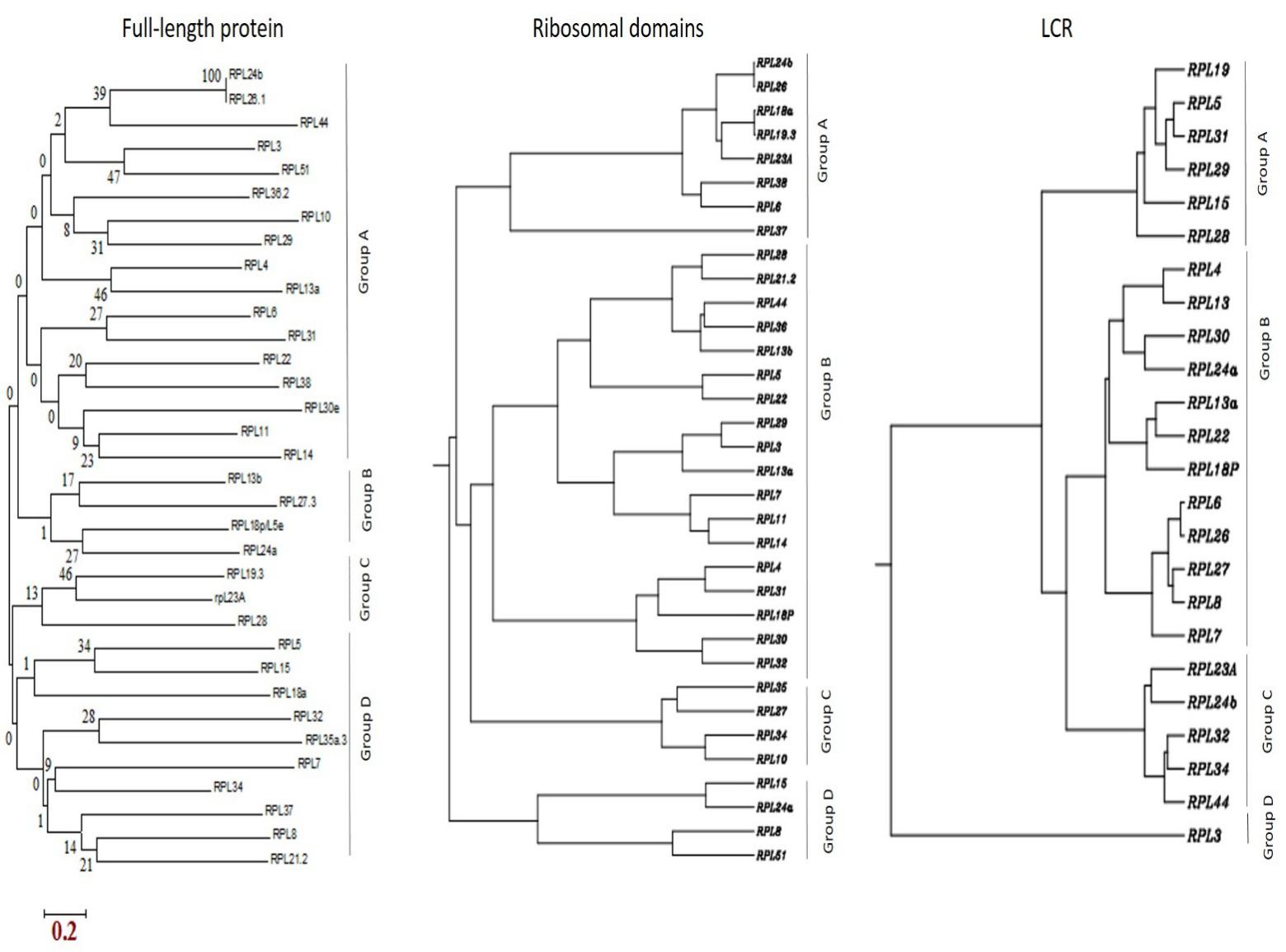
**

**Supplementary Table 1: RPL protein properties**

| **Protein type** | **Length (aa)** | **MW (kDa)** | **p*I*** | **LCR** | **Disordered protein (%)** | **Alpha helix (%)** | **Beta-Strand (%)** | **Ligands** | **Ligand Binding residues** | **GRAVY** | **Specific interactions** |
| --- | --- | --- | --- | --- | --- | --- | --- | --- | --- | --- | --- |
| RPL3 | 390 | 44.4 | 10.73 | one (76-87) | 32 | 12 | 15 |  |  | -0.616 |  |
| RPL4 | 405 | 44.48 | 11.21 | two (126-142) (339-35) | 36 | 45 | 8 |  |  | -0.342 |  |
| RPL5 | 183 | 20.8 | 10.48 | one (135-149) | 16 | 30 | 25 | Ca+2 | ASP30, THR33 | -0.499 |  |
| RPL6 | 223 | 24.6 | 10.8 | 35-56 | 41 | 39 | 16 |  |  | -0.532 | IF-2 |
| RPL7/L12 | 194 | 20.7 | 9.5 | three (106-123, 135-143, 173-186) | 45 | 55 | 6 | two Cu+2 | ALA136, ALA137, LYS138, ILE139, LYS140, ILE141, ILE142, LYS143, GLU161 | -0.230 | EF-Tu, EF-G |
| RPL8 | 262 | 28.2 | 11.5 | 239-260 | 39 | 5 | 39 | Mg+2 | LYS198 | -0.498 |  |
| RPL10 | 234 | 26.5 | 9.3 |  | 15 | 35 | 26 |  |  | -0.573 | L12 interface |
| RPL11 | 167 | 17.7 | 10 |  | 29 | 41 | 22 |  |  | -0.228 | L7/L12 interface, L26 interface |
| RPL13a | 207 | 23.7 | 11.12 | 159-177 | 17 | 57 | 11 | Mg+2 | PRO114, PRO115 | -0.472 | RPL3 interface |
| RPL13b | 204 | 24.05 | 11.55 | two (27-42, 194-208) | 34 | 54 | 1 |  |  | -0.911 |  |
| RPL14 | 135 | 15.3 | 11.01 |  | 15 | 43 | 31 |  |  | -0.313 |  |
| RPL15 | 205 | 24.33 | 12.05 | 58-78 | 32 | 40 | 19 |  |  | -1.042 |  |
| RPL18a | 179 | 21.45 | 11.12 |  | 21 | 26 | 38 |  |  | -0.749 |  |
| RPL18p/L5e | 302 | 34.3 | 9.48 | 15-28, 158-169 | 32 | 42 | 12 |  |  | -0.812 | RPL5 interface |
| RPL19.3 | 204 | 24.14 | 11.95 | two (58-67, 94-107) | 32 | 67 | 3 | Zn+2, Mg+2 | GLU179, ARG180 | -1.058 |  |
| RPL21.2 | 165 | 18.7 | 11.21 |  | 33 | 20 | 35 | Mg+2 | ARG70 | -0.687 |  |
| RPL22 | 172 | 19.48 | 10.9 | 153-163 | 29 | 34 | 16 | Mg+2 | ILE52 | -0.818 |  |
| L23A | 153 | 17.05 | 11.01 | two (24-41, 95-111) | 36 | 34 | 20 | Ca+2 | LYS104 | -0.671 |  |
| RPL24a | 163 | 18.45 | 11.48 | two (116-138, 149-161) | 25 | 68 | 9 |  |  | -1.058 | RPL3, RPL14 interface |
| RPL24b | 158 | 17.47 | 11.71 | three (102-115, 119-133, 135-151) | 45 | 29 | 25 | Mg+2 | LEU118, LYS121, ALA122 | -0.625 |  |
| RPL26.1 | 141 | 15.5 | 11.7 | two (89-102, 127-134) | 44 | 36 | 26 | Mg+2 | LEU105 | -0.606 |  |
| RPL27.3 | 138 | 15.6 | 11.19 | one (107-117) | 29 | 30 | 31 |  | ARG27, PHE29 | -0.377 |  |
| RPL28 | 205 | 23.06 | 10.58 | one (160-178) | 33 | 52 | 8 | Mg+2 | TYR49 | -0.377 |  |
| RPL29 | 198 | 21.08 | 12.93 | three (26-89, 90-110, 155-192) | 40 | 48 | 16 | Mo, FAD, |  | -0.422 | RPL23A interface, Signal recognition particle interaction site, trigger factor interaction site |
| RPL30e | 190 | 20.42 | 10.25 | two (80-94, 103-115) | 49 | 64 | 7 |  |  | -0.124 |  |
| RPL31 | 125 | 14.23 | 10.65 | one (74-88) | 30 | 23 | 35 | Cu+2 | 16VAL, 85ALA, 86ARG, 87ARG, 94ALA | -0.824 |  |
| RPL32 | 134 | 15.67 | 11.2 | one (8-20) | 31 | 24 | 19 |  |  | -0.745 |  |
| RPL34 | 130 | 13.67 | 11.94 | one (96-111) | 32 | 32 | 15 |  |  | -0.913 |  |
| RPL35a.3 | 112 | 12.66 | 11.25 |  | 23 | 10 | 45 | Zn+2, FUC, FAD | 52ARG, 72TRP, 74LYS | -0.699 |  |
| RPL36.2 | 111 | 12.42 | 12.016 |  | 35 | 65 |  |  |  | -0.904 |  |
| RPL37 | 96 | 10.76 | 12.2 |  | 53 | 54 | 3 |  |  | -1.028 |  |
| RPL38 | 75 | 8.7 | 10.6 |  | 16 | 30 | 42 | Zn+2, Mg+2, AMP, CTP | 24ARG, 35LYS, 71LEU | -0.430 |  |
| RPL44 | 106 | 12.13 | 10.94 | one (60-67) | 32 | 32 | 15 | Mg+2 | GLY51 | -1.272 |  |
| RPL51 | 130 | 13.72 | 11.22 |  | 17 | 25 | 33 | Zn+2, Cu+2 | PRO35 | -0.461 |  |

**Supplementary Table 2: Putative promoter analysis of RPL genes**

| **Gene** | **MBS** | **HSE** | **LTR** | **TCA-element** | **TC-rich repeats** | **TGACG motif** | **CGTCA motif** | **BOXW1** | **DRE** | **GARE** | **W box** | **ABRE** | **Motif IIb** | **TGA element** | **AuxR** | **AT rich** | **WUN motif** | **ERE** |
| --- | --- | --- | --- | --- | --- | --- | --- | --- | --- | --- | --- | --- | --- | --- | --- | --- | --- | --- |
| RPL3 |  | * |  | * |  |  |  | ** |  | ** | * |  |  |  |  |  |  |  |
| RPL4 |  |  | * | * |  |  |  | * |  |  | * | * |  | * |  |  |  |  |
| RPL5 | * | * |  |  |  |  |  |  |  | *** |  |  |  |  |  |  |  |  |
| RPL6 | * |  |  |  |  | * | * | * |  |  | * | * |  | ** |  |  |  |  |
| RPL7/L12 | * |  |  |  | ** |  |  |  |  | * |  | * |  |  |  |  |  |  |
| RPL8 | ** | * | * |  |  | ** | ** | * |  |  | * | ***** |  | ** |  |  |  |  |
| RPL10 | *** | * |  | * |  | ****** | ***** |  |  |  |  | * | * | ** |  |  |  |  |
| RPL11 | * |  | * |  |  | * | * | ** |  | * | ** | ** |  |  | * |  |  |  |
| RPL13a | * |  |  |  |  |  |  |  |  |  |  |  |  |  |  |  |  |  |
| RPL13b | ** | ** |  |  | * |  |  |  |  |  |  | **** |  | * |  |  |  |  |
| RPL14 | **** |  |  |  |  |  | ** |  |  | * |  | * |  |  |  |  |  |  |
| RPL15 |  | * |  |  |  | * |  |  |  |  |  | * |  |  |  | ** |  |  |
| RPL18a |  |  |  | ** |  | ** | ** |  | * |  |  | *** | **** | * |  |  |  |  |
| RPL18P | * |  | * | ** |  |  | * | * |  |  | * | ** |  |  |  |  |  |  |
| RPL19.3 |  | * | *** |  | * | ** | ** |  |  |  |  | * |  |  |  |  |  |  |
| RPL21.2 | * | *** | * |  | * | ** | ** |  |  |  |  |  | * |  | * |  |  |  |
| RPL22 | ** |  |  |  | ** |  |  |  |  |  |  |  |  | ** |  |  |  |  |
| RPL23A | *** |  |  |  | * |  | ** | * |  |  | * | * |  |  |  |  | * |  |
| RPL24a |  |  | * |  | ** | * | * |  |  |  |  |  |  | * |  |  |  |  |
| RPL24b | * |  |  |  | * |  |  |  |  | * |  | *** |  |  |  |  |  |  |
| RPL26.1 | *** |  | * |  |  | * |  |  |  |  |  | ** |  | * |  |  |  | * |
| RPL27.3 |  |  | * |  |  |  |  |  |  |  |  |  |  |  |  |  |  | ** |
| RPL28 | * |  |  |  | * | *** | *** |  |  |  |  | ***** |  |  |  | * |  |  |
| RPL29 |  | * |  |  |  |  |  |  |  |  |  | ****** |  | *** |  |  |  |  |
| RPL30e | * |  |  | * |  | * |  | * |  | ** | * |  |  | * | * |  |  |  |
| RPL31 | * |  |  | *** |  | ** | ** |  |  |  |  | * |  | * |  |  |  |  |
| RPL32 |  |  |  | * |  | * | * |  |  |  |  | ** |  |  |  |  |  |  |
| RPL34 | * | * |  | * | ** |  |  | * |  |  | * | * |  |  |  |  |  |  |
| RPL35a.3 |  | * | * | ** | * | * | * |  |  |  |  | **** |  |  |  |  |  |  |
| RPL36.2 |  |  |  |  | * | * | * |  |  |  |  | *** |  |  |  |  |  |  |
| RPL37 |  |  |  |  |  |  |  |  |  | * |  |  |  |  |  |  |  |  |
| RPL38 | *** |  |  |  |  | * |  |  |  |  |  | * |  | * |  |  |  |  |
| RPL44 |  |  |  |  | * | ** | ** |  |  |  |  | ** | * | * |  |  |  |  |
| RPL51 |  | * | * |  | ** |  |  |  |  | * |  |  |  |  |  |  |  |  |

The presence or *cis-*regulatory elements were analyzed using PlantCARE database. MBS (MYB binding site), DRE (Drought Responsive Element), W-box (WRKY transcription factor binding elements), HSE (Heat Stress Elements), LTR (Low Temperature Responsiveness), TCA-element (Salicylic Acid responsiveness), TC-rich repeats (Defence and stress responsiveness), TGACG and CGTCA -motif (MeJa responsiveness), BOXW1 (Fungal elicitor-responsive element), ERE (Ethylene Responsive Element), GARE (Gibberellin-Responsive Element), WUN-motif (Wound responsiveness), ABRE (ABA Responsive Element), TGA-element, Motif IIb (Auxin-responsive element), AuxR and TGA-element (Auxin Responsiveness).

**Supplementary Table 3: Primers used in gene expression studies**

| **Primer Name** | **Sequence** |
| --- | --- |
| L3 RT FP | TGGACTTGTGGCCTATGTGA |
| L3 RT RP | CCGGCATCGCTATCATACTT |
| L4 RT FP | AAGAAGCTCGACGAGGTGTA |
| L4 RT RP | CCACATTCTTCAGAGGGTTC |
| L5 RT FP | GATCTTGGCATCAAGTACGAC |
| L5 RT RP | GACACCCTCATACTTGACCTG |
| L6 RT FP | GTTCCTCAAGCAGCTCAAAT |
| L6 RT RP | CTTCTGCTTCTTGTCCCTAGA |
| L7 RT FP | TACCCAAACCTGAAGAGTGTC |
| L7 RT RP | GACAGTCATGATCTCGTGGA |
| L8 RT FP | ACTACGCCATCGTCATCAG |
| L8 RT RP | GGTACTTGTGGTAGGCGTTT |
| L10 RT FP | AGAAGAAGCCTGGATTAGAGC |
| L10 RT RP | ATATCCTGCTGGAGGACTTG |
| L11 RT FP | AAGAAGATCGGTGAGGACATC |
| L11 RT RP | TCTTGACCTTCTTCCTGTCC |
| L12 RT FP | GCTCATTTGTACAGCACAGAG |
| L12 RT RP | TTGGTTCAGTCTGAGAAGGAG |
| L13a RT FP | GAACTACCACGACACCATCAG |
| L13a RT RP | GGGGCCAAAATATCTATCTG |
| L13b RT FP | AAGCACTGGCAGAACTATGTC |
| L13b RT RP | CCCTCGACTTCATGTTGTACT |
| L14 RT FP | GTGAACTACGGCAAGGACTAC |
| L14 RT RP | TAACATCAGCCTCCTCCATAG |
| L15 RT FP | ACAAGTACGTGTCGGAGCTAT |
| L15 RT RP | GACACGGTAAACCACATAACC |
| L18a RT FP | TCCAAGTTCTGGTACTTCCTG |
| L18a RT RP | GTTGTGGTAACCTGTTCTGCT |
| L18p RT FP | TGGGGAGGACTACTATGTTGA |
| L18p RT RP | AAACCTCTTGTCACTGTGAGG |
| L19.3 RT FP | AGTATCGTGAGGCCAAGAAG |
| L19.3 RT RP | CTTAGCCTCAAACTGGTCAGA |
| L21.2 RT FP | CTGAGGAAGATCAAGAACGAC |
| L21.2 RT RP | AACCACCCTTGAGATCATTG |
| L22 RT FP | GAGGTGAAAGGTCTGGATGTT |
| L22 RT RP | TCACTGGTTCTTCCTTCTCTG |
| L23A RT FP | GACCAAAGACCCTGAAGAAGG |
| L23A RT RP | ACGATGAAGACAAGGGTGTTG |
| L24b RT FP | GTTGGTGCTACACTGGAAGTT |
| L24b RT RP | CCTTCGACTGTGTCTTCTGAG |
| L26.1 RT FP | ACAAGTACAACGTGGTGAGG |
| L26.1 RT RP | GTCCTTGTCGAGCTTGAGTT |
| L27.3 RT FP | CTTCCTCAAGCTCGTCAACT |
| L27.3 RT RP | CTTGGTGAAGAACCACCTGT |
| L28 RT FP | TAGACGAATACCTCCTGAAGA |
| L28 RT RP | AAACCCTGTTCGATCTTAGTC |
| L29 RT FP | CCCAACAAGCTCTCCAATATA |
| L29 RT RP | AGAAACAGAAGCATTCCCTG |
| L30e RT FP | GAGCAAGAAGAAGAACAAGTC |
| L30e RT RP | GCTTCATCCATATCTTTTCCG |
| L31 RT FP | TCAAGGAGATCAGGAAGTTTG |
| L31 RT RP | AACAGTGACCAGAGAGTAGAG |
| L32 RT FP | GCCTAATATTGGCTATGGTTC |
| L32 RT RP | CTTCTTCGTTGAGACATTGTG |
| L34 RT FP | GAAGAAGATCCAGGGAATTCC |
| L34 RT RP | CACAATCTTCTGCTCTTCAAC |
| L35a.3 RT FP | CTACGTCTACAAGGCCAAG |
| L35a.3 RT RP | TGCTGGGGTACATGAAGA |
| L36.2 RT FP | GGAAAAGTACCAAGAGAGTGA |
| L36.2 RT RP | CTTCTTCTTTGCTCTCTTGTG |
| L37 RT FP | CTTCCACCTGCAGAAGAG |
| L37 RT RP | CCCCTCTCTGAAGTTACTCT |
| L38 RT FP | CACGAGATCAAGGACTTCC |
| L38 RT RP | AAAGGTGGATGAAATGTAGGC |
| L44 RT FP | AAGAAGACCTACTGCAAGAAC |
| L44 RT RP | CCTTACCCTTCTTGTACTGAG |
| L51 RT FP | GTGACAGAGTTAGTCCGTGGA |
| L51 RT RP | TCTCAGCTTCACCACTTTCCT |

**Supplementary Table 4:** RPL genes with fold levels ≥2 on the log_2_ scale were considered as up-regulated. The table details the RPL genes expressing in each tissue specifically

| **Embryo** | **Endosperm** | **Plumules** | **Radicles** | **6 d s** | **6 d r** | **Roots** | **Shoots** | **Leaves** | **Panicles** | **Grains** | **Flowers** | **Root-shoot transition** |
| --- | --- | --- | --- | --- | --- | --- | --- | --- | --- | --- | --- | --- |
| L5 | L5 | L5 | L5 | L5 | L5 | L5 | L5 | L5 | L5 | L5 |  |  |
|  |  | L4 | L4 |  | L4 | L4 | L4 | L4 |  |  | L4 |  |
|  |  | L6 | L6 | L6 | L6 |  | L6 | L6 |  | L6 |  |  |
| L7 | L7 | L7 |  |  | L7 | L7 | L7 |  | L7 | L7 |  |  |
| L8 | L8 | L8 |  |  |  | L8 | L8 |  |  | L8 |  |  |
|  |  |  |  |  |  |  | L10 |  |  |  |  |  |
|  |  |  |  |  |  | L11 | L11 | L11 | L11 |  |  |  |
|  |  | L12 |  |  |  |  |  |  |  |  |  |  |
|  |  | L13a | L13a |  | L13a | L13a | L13a | L13a | L13a | L13a | L13a | L13a |
|  |  | L13b |  |  |  |  | L13b |  |  |  |  |  |
|  | L14 | L14 |  |  | L14 | L14 | L14 | L14 | L14 | L14 | L14 | L14 |
|  |  |  |  |  | L15 |  |  |  |  |  |  |  |
|  | L18a |  |  |  |  | L18a | L18a | L18a |  | L18a |  |  |
| L18P | L18P | L18P |  |  |  |  | L18P | L18P |  | L18P |  |  |
| L19.3 | L19.3 | L19.3 | L19.3 | L19.3 |  | L19.3 | L19.3 | L19.3 | L19.3 | L19.3 | L19.3 | L19.3 |
| L21.2 | L21.2 |  |  |  |  | L21.2 | L21.2 | L21.2 | L21.2 | L21.2 |  |  |
| L22 | L22 |  |  | L22 |  | L22 | L22 | L22 | L22 | L22 | L22 | L22 |
|  | L23 | L23 | L23 |  | L23 | L23 | L23 | L23 |  | L23 | L23 |  |
| L24 | L24 | L24 | L24 | L24 | L24 | L24 | L24 | L24 | L24 | L24 | L24 | L24 |
|  | L24b |  | L24b |  |  |  | L24b |  |  |  |  |  |
|  |  |  |  |  |  |  | L26 | L26 | L26 | L26 | L26 |  |
|  | L27 |  |  |  |  |  | L27 |  |  |  | L27 |  |
|  |  |  |  |  |  |  |  |  |  |  | L29 |  |
|  |  |  |  |  |  | L30 | L30 |  |  |  |  |  |
| L31 | L31 |  | L31 | L31 | L31 | L31 | L31 | L31 | L31 |  |  | L31 |
|  |  |  |  |  |  | L32 | L32 | L32 | L32 |  | L32 | L32 |
| L34 | L34 |  | L34 | L34 | L34 | L34 | L34 | L34 | L34 | L34 | L34 |  |
| L35 |  |  | L35 |  |  | L35 | L35 | L35 | L35 | L35 |  | L35 |
|  |  |  |  | L36 |  |  | L36 | L36 | L36 |  |  |  |
|  |  |  |  |  |  | L37 | L37 |  | L37 |  |  |  |
| L38 |  |  | L38 |  |  | L38 | L38 | L38 | L38 |  |  |  |
|  | L44 |  | L44 | L44 |  | L44 | L44 | L44 | L44 | L44 |  |  |
|  |  |  | L51 | L51 | L51 | L51 | L51 |  | L51 |  |  |  |

**Supplementary Table 5: Up-regulation of large subunit ribosomal genes at different time intervals after stress treatments in shoot and root tissues**

**Supplementary Table 5a: Immediate-early (IE) genes up-regulation in shoots**

| **5 min after treatment** | **Genes Upregulated in shoots** | **3 h after treatment** | **Genes upregulated in shoots** |
| --- | --- | --- | --- |
| Cold-5 min, H2O2-5 min, Heat-5 min, MeJa-5 min, SA-5 min | RPL13a | Cold-3 h Heat-3 h MeJa-3 h SA-3 h | RPL8 RPL6 RPL10 RPL12 |
| Cold-5 min Heat-5 min MeJa-5 min SA-5 min | RPL11 RPL23 RPL8 RPL51 RPL6 RPL10 RPL12 RPL7 | Cold-3 h MeJa-3 h SA-3 h | RPL44 RPL19.3 RPL5 RPL35 RPL31 RPL13b RPL7 RPL32 RPL37 |
| Cold-5 min H2O2-5 min MeJa-5 min SA-5 min | RPL32 RPL37 | Heat-3 h MeJa-3 h SA-3 h | RPL11 |
| Cold-5 min H2O2-5 min Heat-5 min SA-5 min | RPL28 | Cold-3 h Heat-3 h MeJa-3 h | RPL4 |
| Cold-5 min MeJa-5 min SA-5 min | RPL44 RPL19.3 RPL35 RPL13b RPL27 | Cold-3 h H2O2-3 h MeJa-3 h | RPL28 |
| Cold-5 min H2O2-5 min SA-5 min | RPL38 | Cold-3 h Heat-3 h SA-3 h | RPL23 |
| MeJa-5 min SA-5 min | RPL5 RPL22 | MeJa-3 h SA-3 h | RPL38 RPL51 RPL27 |
| Cold-5 min MeJa-5 min | RPL18P | Cold-3 h MeJa-3 h | RPL18P |
| Cold-5 min SA-5 min | RPL24 | Cold-3 h SA-3 h | RPL30 RPL13a RPL24 |
| Cold-5 min Heat-5 min | RPL18a 5 | H2O2-3 h SA-3 h | RPL22 |
| MeJa-5 min | RPL24a | MeJa-3 h | RPL24a RPL34 RPL36 |
| SA-5 min | RPL26 | SA-3 h | RPL29 RPL26 |
| Cold-5 min | RPL30 RPL31 RPL15 | Cold-3 h | RPL15 |
| H2O2-5 min | RPL4 | H2O2-3 h | RPL18a |

**Supplementary Table 5b: Early (E) genes up-regulation in shoots**

| **6 h after treatment** | **Genes upregulated in shoots** | **12 h after treatment** | **Genes upregulated in shoots** |
| --- | --- | --- | --- |
| Cold-6 h H2O2-6 h Heat-6 h MeJa-6 h SA-6 h | RPL28 | Cold-12 h Heat-12 h MeJa-12 h SA-12 h | RPL23 RPL12 |
| Cold-6 h Heat-6 h MeJa-6 h SA-6 h | RPL11 RPL23 RPL10 RPL12 RPL7 | Cold-12 h H2O2-12 h MeJa-12 h SA-12 h | RPL13a RPL19.3 RPL7 RPL32 |
| Cold-6 h H2O2-6 h MeJa-6 h SA-6 h | RPL19.3 | H2O2-12 h Heat-12 h MeJa-12 h SA-12 h | RPL28 |
| Cold-6 h MeJa-6 h SA-6 h | RPL35 RPL8 RPL13b RPL27 | Cold-12 h H2O2-12 h Heat-12 h MeJa-12 h | RPL6 |
| H2O2-6 h MeJa-6 h SA-6 h | RPL44 RPL22 | Cold-12 h MeJa-12 h SA-12 h | RPL35 RPL8 |
| Cold-6 h Heat-6 h SA-6 h | RPL24 | Heat-12 h MeJa-12 h SA-12 h | RPL11 RPL10 |
| MeJa-6 h SA-6 h | RPL30 RPL26 | H2O2-12 h MeJa-12 h SA-12 h | RPL44 RPL31 RPL22 RPL5 RPL30 RPL34 RPL38 RPL51 RPL37 |
| Cold-6 h MeJa-6 h | RPL32 | Cold-12 h H2O2-12 h MeJa-12 h | RPL4 |
| H2O2-6 h MeJa-6 h | RPL29 | MeJa-12 h SA-12 h | RPL18P RPL13b RPL27 RPL26 |
| Cold-6 h SA-6 h | RPL18P RPL4 | H2O2-12 h MeJa-12 h | RPL29 |
| Cold-6 h Heat-6 h | RPL6 | H2O2-12 h SA-12 h | RPL18a |
| Cold-6 h H2O2-6 h | RPL37 | MeJa-12 h | RPL24a |
| MeJa-6 h | RPL24a | SA-12 h | RPL24 |
| SA-6 h | 6h RPL24b | H2O2-12 h | RPL14 RPL21.2 |
| Cold-6 h | RPL31 |  |  |
| H2O2-6 h | RPL36 RPL51 RPL18a |  |  |

**Supplementary Table 5c: Late (L) genes up-regulation in shoots**

| **24 h after treatment** | **Genes upregulated in shoots** | **60 h after treatment** | **Genes upregulated in shoots** |
| --- | --- | --- | --- |
| Cold-24 h H2O2-24 h Heat-24 h MeJa-24 h SA-24 h | RPL28 | Cold-60 h H2O2-60 h MeJa-60 h SA-60 h | RPL31 |
| Cold-24 h Heat-24 h MeJa-24 h SA-24 h | RPL18P RPL23 RPL10 RPL12 | Cold-60 h MeJa-60 h SA-60 h | RPL44 RPL19.3 RPL30 RPL35 RPL13a RPL18P RPL4 RPL38 RPL23 RPL8 RPL12 RPL13b RPL7 RPL32 |
| Cold-24 h H2O2-24 h MeJa-24 h SA-24 h | RPL5 RPL31 | H2O2-60 h MeJa-60 h SA-60 h | RPL5 RPL22 |
| Cold-24 h MeJa-24 h SA-24 h | RPL44 RPL19.3 RPL35 RPL8 RPL13b RPL7 RPL32 | Cold-60 h H2O2-60 h SA-60 h | RPL28 RPL18a |
| Heat-24 h MeJa-24 h SA-24 h | RPL11 | MeJa-60 h SA-60 h | RPL11 RPL26 RPL51 RPL10 RPL27 |
| H2O2-24 h MeJa-24 h SA-24 h | RPL22 | Cold-60 h MeJa-60 h | RPL34 RPL15 |
| Cold-24 h Heat-24 h SA-24 h | RPL24 | Cold-60 h SA-60 h | RPL6 RPL24 |
| Cold-24 h H2O2-24 h SA-24 h | RPL18a | MeJa-60 h | RPL24a |
| MeJa-24 h SA-24 h | RPL30 RPL13a RPL4 RPL38 RPL26 RPL51 RPL27 | SA-60 h | RPL29 |
| Cold-24 h MeJa-24 h | RPL34 RPL15 RPL37 | Cold-60 h | RPL37 |
| Cold-24 h Heat-24 h | RPL6 |  |  |
| MeJa-24 h | RPL24a |  |  |
| Cold-24 h | RPL24b |  |  |

**Supplementary Table 5d: Immediate-early (IE) genes up-regulation in roots**

| **5 min after treatments** | **Genes upregulated in roots** | **3 h after treatment** | **Genes upregulated in roots** |
| --- | --- | --- | --- |
| Cold-5 min Heat-5 min MeJa-5 min SA-5 min | RPL6 | Cold-3 h Heat-3 h MeJa-3 h SA-3 h | RPL18P RPL23 RPL24 RPL7 RPL32 |
| Cold-5 min MeJa-5 min SA-5 min | RPL28 RPL19.3 RPL35 RPL31 RPL18P RPL21.2 RPL38 RPL24 RPL7 RPL32 RPL37 | Cold-3 h MeJa-3 h SA-3 h | RPL19.3 RPL35 RPL31 RPL21.2 RPL38 RPL51 RPL37 |
| H2O2-5 min MeJa-5 min SA-5 min | RPL8 | Heat-3 h MeJa-3 h SA-3 h | RPL14 RPL8 RPL12 |
| Cold-5 min Heat-5 min MeJa-5 min | RPL23 | Cold-3 h Heat-3 h SA-3 h | RPL11 RPL6 |
| Cold-5 min H2O2-5 min MeJa-5 min | RPL51 | MeJa-3 h SA-3 h | RPL26 RPL13b |
| MeJa-5 min SA-5 min | RPL5 RPL34 RPL14 RPL26 RPL12 RPL13b | Cold-3 h SA-3 h | RPL28 |
| Cold-5 min MeJa-5 min | RPL36 | H2O2-3 h SA-3 h | RPL5 RPL34 |
| Cold-5 min | RPL29 RPL10 | Cold-3 h H2O2-3 h | RPL24b |
| H2O2-5 min | RPL18a | Cold-3 h | RPL44 RPL29 RPL36 |
|  |  | Heat-3 h | RPL10 RPL22 |
|  |  | H2O2-3 h | RPL15 RPL18a |

**Supplementary Table 5e: Early (E) genes up-regulation in roots**

| **6 h after treatment** | **Genes upregulated in roots** | **12 h after treatment** | **Genes upregulated in roots** |
| --- | --- | --- | --- |
| Cold-6 h H2O2-6 h Heat-6 h MeJa-6 h SA-6 h | RPL7 | Cold-12 h H2O2-12 h Heat-12 h MeJa-12 h SA-12 h | RPL18P RPL23 RPL6 |
| Cold-6 h Heat-6 h MeJa-6 h SA-6 h | RPL12 | Cold-12 h Heat-12 h MeJa-12 h SA-12 h | RPL32 |
| Cold-6 h H2O2-6 h MeJa-6 h SA-6 h | RPL35 RPL31 RPL21.2 RPL38 RPL51 RPL32 RPL37 | Cold-12 h H2O2-12 h MeJa-12 h SA-12 h | RPL26 RPL51 RPL13b |
| H2O2-6 h Heat-6 h MeJa-6 h SA-6 h | RPL18P | Cold-12 h MeJa-12 h SA-12 h | RPL35 RPL14 RPL38 RPL8 RPL37 |
| Cold-6 h H2O2-6 h Heat-6 h MeJa-6 h | RPL23 RPL6 | Heat-12 h MeJa-12 h SA-12 h | RPL12 RPL24 RPL7 |
| Cold-6 h MeJa-6 h SA-6 h | RPL28 RPL8 | H2O2-12 h MeJa-12 h SA-12 h | RPL34 |
| H2O2-6 h MeJa-6 h SA-6 h | RPL19.3 | Cold-12 h Heat-12 h SA-12 h | RPL28 |
| MeJa-6 h SA-6 h | RPL14 RPL13b RPL24 | MeJa-12 h SA-12 h | RPL19.3 RPL5 RPL31 RPL21.2 |
| H2O2-6 h MeJa-6 h | RPL26 | H2O2-12 h MeJa-12 h | RPL36 |
| Cold-6 h H2O2-6 h | RPL29 RPL24b RPL36 | Cold-12 h SA-12 h | RPL10 |
| H2O2-6 h Heat-6 h | RPL22 | H2O2-12 h SA-12 h | RPL4 |
| Cold-6 h | RPL11 | Cold-12 h H2O2-12 h | RPL29 |
| H2O2-6 h | RPL44 RPL30 RPL13a RPL34 | H2O2-12 h Heat-12 h | RPL27 |
|  |  | Cold-12 h | RPL11 |
|  |  | Heat-12 h | RPL22 |
|  |  | H2O2-12 h | RPL30 RPL13a RPL24b |

**Supplementary Table 5f: Late (L) genes up-regulation in roots**

| **24 h after treatment** | **Genes upregulated in roots** | **60 h after treatment** | **Genes upregulated in roots** |
| --- | --- | --- | --- |
| Cold-24 h Heat-24 h MeJa-24 h SA-24 h | RPL18P RPL23 | Cold-60 h H2O2-60 h MeJa-60 h SA-60 h | RPL35 RPL38 RPL26 RPL32 RPL37 |
| Cold-24 h H2O2-24 h MeJa-24 h SA-24 h | RPL35 RPL14 RPL38 RPL36 RPL51 RPL32 RPL37 | Cold-60 h MeJa-60 h SA-60 h | RPL18P RPL19.3 RPL23 |
| Cold-24 h MeJa-24 h SA-24 h | RPL31 RPL21.2 RPL13b RPL7 | H2O2-60 h MeJa-60 h SA-60 h | RPL14 RPL36 |
| Heat-24 h MeJa-24 h SA-24 h | RPL12 | Cold-60 h H2O2-60 h SA-60 h | RPL51 |
| H2O2-24 h MeJa-24 h SA-24 h | RPL26 RPL18a | MeJa-60 h SA-60 h | RPL31 RPL13b RPL24 RPL21.2 RPL8 RPL10 RPL12 RPL7 |
| MeJa-24 h SA-24 h | RPL19.3 RPL8 RPL10 RPL24 | Cold-60 h SA-60 h | RPL11 RPL28 RPL6 |
| Cold-24 h SA-24 h | RPL28 RPL11 | H2O2-60 h SA-60 h | RPL34 RPL18a |
| H2O2-24 h SA-24 h | RPL34 | SA-60 h | RPL5 RPL4 |
| Cold-24 h Heat-24 h | RPL6 | Cold-60 h | RPL29 |
| H2O2-24 h Heat-24 h | RPL22 | H2O2-60 h | RPL13a RPL27 RPL22 RPL30 RPL24b |
| SA-24 h | RPL5 |  |  |
| Cold-24 h | RPL29 |  |  |
| H2O2-24 h | RPL30 RPL13a RPL24b RPL27 |  |  |

**Supplementary Table 6: Down-regulation of large subunit ribosomal genes at different time intervals after stress treatments in shoot and root tissues**

**Supplementary Table-6a: IE genes down-regulation in shoots**

| **3 h after treatment** | **Genes downregulated in shoots** | **6 h after treatment** | **Genes downregulated in shoots** |
| --- | --- | --- | --- |
| Cold-3 h H2O2-3 h Heat-3 h MeJa-3 h SA-3 h | RPL14 | Cold-6 h H2O2-6 h Heat-6 h MeJa-6 h SA-6 h | RPL31 RPL14 RPL5 RPL34 RPL38 |
| Cold-3 h H2O2-3 h Heat-3 h SA-3 h | RPL34 | Cold-6 h Heat-6 h MeJa-6 h SA-6 h | RPL51 |
| Cold-3 h Heat-3 h MeJa-3 h | RPL22 | Cold-6 h H2O2-6 h Heat-6 h MeJa-6 h | RPL13a |
| H2O2-3 h Heat-3 h MeJa-3 h | RPL13a RPL29 RPL30 | H2O2-6 h MeJa-6 h SA-6 h | RPL6 |
| Cold-3 h Heat-3 h SA-3 h | RPL24b RPL18a | Cold-6 h H2O2-6 h MeJa-6 h | RPL15 |
| Cold-3 h H2O2-3 h Heat-3 h | RPL21.2 RPL38 RPL51 | H2O2-6 h Heat-6 h MeJa-6 h | RPL4 |
| Heat-3 h MeJa-3 h | RPL26 | Cold-6 h Heat-6 h SA-6 h | RPL18a |
| H2O2-3 h MeJa-3 h | RPL24b RPL15 | H2O2-6 h Heat-6 h SA-6 h | RPL32 |
| Heat-3 h SA-3 h | RPL18P RPL28 | Cold-6 h H2O2-6 h Heat-6 h | RPL30 RPL21.2 |
| H2O2-3 h SA-3 h | RPL4 | MeJa-6 h SA-6 h | RPL37 |
| Cold-3 h Heat-3 h | RPL27 | Heat-6 h MeJa-6 h | RPL18P RPL36 |
| Cold-3 h H2O2-3 h | RPL11 | H2O2-6 h MeJa-6 h | RPL24b |
| H2O2-3 h Heat-3 h | RPL44 RPL31 RPL36 RPL5 RPL7 RPL32 | Heat-6 h SA-6 h | RPL29 |
| MeJa-3 h | RPL23 | Cold-6 h Heat-6 h | RPL44 RPL22 RPL24b |
| Heat-3 h | RPL35 RPL24 RPL13b | Heat-6 h | RPL35 RPL13b RPL27 RPL8 RPL26 |
| H2O2-3 h | RPL26 RPL27 RPL24 RPL19.3 23 RPL6 RPL10 RPL12 RPL37 | H2O2-6 h | RPL11 RPL26 RPL27 RPL24 RPL23 RPL10 RPL12 RPL7 |

**Supplementary Table 6b: Early (E) genes down-regulation in shoots**

| **12 h after treatment** | **Genes downregulated in shoots** | **24 after treatment** | **Genes downregulated in shoots** |
| --- | --- | --- | --- |
| Cold-12 h Heat-12 h MeJa-12 h SA-12 h | RPL14 | Cold-24 h H2O2-24 h Heat-24 h MeJa-24 h SA-24 h | RPL14 |
| Cold-12 h H2O2-12 h MeJa-12 h | RPL15 | H2O2-24 h Heat-24 h MeJa-24 h SA-24 h | RPL29 |
| H2O2-12 h Heat-12 h MeJa-12 h | RPL36 | H2O2-24 h Heat-24 h MeJa-24 h | RPL36 |
| Cold-12 h Heat-12 h SA-12 h | RPL24b | H2O2-24 h Heat-24 h SA-24 h | RPL34 |
| Cold-12 h H2O2-12 h Heat-12 h | RPL44 | Cold-24 h H2O2-24 h Heat-24 h | RPL13a RPL30 RPL4 RPL21.2 RPL38 RPL51 |
| H2O2-12 h MeJa-12 h | RPL24b | H2O2-24 h MeJa-24 h | RPL24b |
| Heat-12 h SA-12 h | RPL29 RPL4 | Heat-24 h SA-24 h | RPL24b |
| Cold-12 h Heat-12 h | RPL18P RPL24 RPL13b RPL27 RPL22 RPL5 RPL30 RPL21.2 RPL34 RPL38 RPL51 RPL18a | H2O2-24 h SA-24 h | RPL6 RPL37 |
| Cold-12 h H2O2-12 h | RPL11 RPL10 | Cold-24 h Heat-24 h | RPL27 RPL22 |
| SA-12 h | RPL6 | Cold-24 h H2O2-24 h | RPL11 |
| Cold-12 h | RPL28 RPL37 | H2O2-24 h Heat-24 h | RPL44 RPL7 RPL32 |
| Heat-12 h | RPL35 RPL13a RPL31 RPL8 RPL26 RPL7 RPL32 | Heat-24 h | RPL35 RPL31 RPL13b RPL5 RPL8 RPL26 RPL18a |
| H2O2-12 h | RPL26 RPL27 RPL24 RPL23 RPL12 | H2O2-24 h | RPL26 RPL27 RPL24 RPL19.3 RPL23 RPL15 RPL10 RPL12 |

**Supplementary Table 6c: Late (L) genes down-regulation in shoots**

| **60 h after treatment** | **Genes downregulated in shoots** | **5 min after treatment** | **Genes downregulated in roots** |
| --- | --- | --- | --- |
| Cold-60 h H2O2-60 h MeJa-60 h SA-60 h | RPL14 | Cold-5 min H2O2-5 min Heat-5 min MeJa-5 min SA-5 min | RPL13a |
| H2O2-60 h MeJa-60 h SA-60 h | RPL37 | Cold-5 min Heat-5 min MeJa-5 min SA-5 min | RPL18a |
| H2O2-60 h MeJa-60 h | RPL29 RPL36 24b | H2O2-5 min Heat-5 min MeJa-5 min SA-5 min | RPL11 RPL29 RPL30 RPL4 RPL10 |
| Cold-60 h SA-60 h | RPL24b | Cold-5 min H2O2-5 min Heat-5 min MeJa-5 min | RPL44 RPL22 |
| H2O2-60 h SA-60 h | RPL34 | Cold-5 min H2O2-5 min Heat-5 min SA-5 min | RPL24b |
| Cold-60 h H2O2-60 h | RPL11 RPL21.2 RPL51 RPL10 | H2O2-5 min Heat-5 min MeJa-5 min | RPL27 |
| MeJa-60 h | RPL28 | H2O2-5 min Heat-5 min SA-5 min | RPL36 |
| Cold-60 h | RPL5 RPL27 RPL22 | Cold-5 min H2O2-5 min Heat-5 min | RPL18P RPL14 RPL13b RPL34 RPL26 RPL12 |
| H2O2-60 h | RPL44 RPL19.3 RPL30 RPL13a RPL26 RPL4 RPL38 RPL23 RPL15 RPL27 RPL6 RPL12 RPL7 RPL32 RPL24 | Heat-5 min MeJa-5 min | RPL28 |
|  |  | H2O2-5 min MeJa-5 min | RPL15 |
|  |  | Heat-5 min SA-5 min | RPL51 |
|  |  | H2O2-5 min SA-5 min | RPL23 |
|  |  | Cold-5 min Heat-5 min | RPL8 |
|  |  | H2O2-5 min Heat-5 min | RPL35 RPL31 RPL5 RPL21.2 RPL38 RPL7 RPL32 |
|  |  | Heat-5 min | RPL24 |
|  |  | H2O2-5 min | RPL19.3 RPL6 RPL37 |

**Supplementary Table 6d: IE genes down-regulation in roots**

| **5 min after treatment** | **Genes downregulated in roots** | **3 h after treatment** | **Genes downregulated in roots** |
| --- | --- | --- | --- |
| Cold-5 min H2O2-5 min Heat-5 min MeJa-5 min SA-5 min | RPL13a | Cold-3 h H2O2-3 h Heat-3 h MeJa-3 h SA-3 h | RPL13a |
| Cold-5 min Heat-5 min MeJa-5 min SA-5 min | RPL18a | Cold-3 h Heat-3 h MeJa-3 h SA-3 h | RPL18a |
| H2O2-5 min Heat-5 min MeJa-5 min SA-5 min | RPL11 RPL29 RPL30 RPL4 RPL10 | Cold-3 h H2O2-3 h MeJa-3 h SA-3 h | RPL10 |
| Cold-5 min H2O2-5 min Heat-5 min MeJa-5 min | RPL44 RPL22 | H2O2-3 h Heat-3 h MeJa-3 h SA-3 h | RPL30 RPL29 RPL4 RPL36 |
| Cold-5 min H2O2-5 min Heat-5 min SA-5 min | RPL24b | Cold-3 h Heat-3 h MeJa-3 h | RPL34 |
| H2O2-5 min Heat-5 min MeJa-5 min | RPL27 | Cold-3 h H2O2-3 h MeJa-3 h | RPL22 |
| H2O2-5 min Heat-5 min SA-5 min | RPL36 | H2O2-3 h Heat-3 h MeJa-3 h | RPL44 RPL27 |
| Cold-5 min H2O2-5 min Heat-5 min | RPL18P RPL14 RPL13b RPL34 RPL26 RPL12 | Cold-3 h H2O2-3 h Heat-3 h | RPL26 RPL13b |
| Heat-5 min MeJa-5 min | RPL28 | Heat-3 h MeJa-3 h | RPL5 |
| H2O2-5 min MeJa-5 min | RPL15 | H2O2-3 h MeJa-3 h | RPL11 RPL6 |
| Heat-5 min SA-5 min | RPL51 | Heat-3 h SA-3 h | RPL24b |
| H2O2-5 min SA-5 min | RPL23 | Cold-3 h H2O2-3 h | RPL18P RPL14 RPL8 RPL12 |
| Cold-5 min Heat-5 min | RPL8 | H2O2-3 h Heat-3 h | RPL35 RPL31 RPL21.2 RPL38 RPL51 |
| H2O2-5 min Heat-5 min | RPL35 RPL31 RPL5 RPL21.2 RPL38 RPL7 RPL32 | MeJa-3 h | RPL15 |
| Heat-5 min | RPL24 | Heat-3 h | RPL28 |
| H2O2-5 min | RPL19.3 RPL6 RPL37 | H2O2-3 h | RPL19.3 RPL23 RPL7 RPL32 RPL37 |

**Supplementary Table 6e: Early (E) genes down-regulation in roots**

| **6 h after treatment** | **Genes downregulated in roots** | **12 h after treatment** | **Genes downregulated in roots** |
| --- | --- | --- | --- |
| Cold-6 h H2O2-6 h Heat-6 h MeJa-6 h SA-6 h | RPL10 RPL18a | Cold-12 h H2O2-12 h Heat-12 h MeJa-12 h SA-12 h | RPL18a |
| Cold-6 h Heat-6 h MeJa-6 h SA-6 h | RPL13a RPL34 | Cold-12 h Heat-12 h MeJa-12 h SA-12 h | RPL13a |
| H2O2-6 h Heat-6 h MeJa-6 h SA-6 h | RPL11 RPL5 RPL4 | H2O2-12 h Heat-12 h MeJa-12 h SA-12 h | RPL11 |
| Heat-6 h MeJa-6 h SA-6 h | RPL29 RPL36 RPL30 | Cold-12 h H2O2-12 h Heat-12 h MeJa-12 h | RPL44 |
| Cold-6 h Heat-6 h MeJa-6 h | RPL44 | Heat-12 h MeJa-12 h SA-12 h | RPL30 RPL29 |
| H2O2-6 h Heat-6 h MeJa-6 h | RPL27 | Cold-12 h H2O2-12 h MeJa-12 h | RPL22 |
| Cold-6 h Heat-6 h SA-6 h | RPL26 | H2O2-12 h Heat-12 h MeJa-12 h | RPL10 |
| Cold-6 h H2O2-6 h Heat-6 h | RPL14 RPL13b | Cold-12 h Heat-12 h SA-12 h | RPL24b RPL36 |
| Cold-6 h MeJa-6 h | RPL22 | Cold-12 h H2O2-12 h Heat-12 h | RPL31 RPL21.2 |
| H2O2-6 h MeJa-6 h | RPL15 | Heat-12 h MeJa-12 h | RPL4 |
| Heat-6 h SA-6 h | RPL24b | H2O2-12 h MeJa-12 h | RPL15 |
| Cold-6 h Heat-6 h | RPL24 | Cold-12 h Heat-12 h | RPL34 |
| H2O2-6 h Heat-6 h | RPL8 | Cold-12 h H2O2-12 h | RPL19.3 RPL12 RPL7 |
| SA-6 h | RPL23 RPL6 | H2O2-12 h Heat-12 h | RPL5 RPL35 RPL14 RPL38 RPL8 |
| Cold-6 h | RPL18P RPL19.3 | MeJa-12 h | RPL28 RPL27 |
| Heat-6 h | RPL35 RPL31 RPL28 RPL21.2 RPL38 RPL51 RPL32 | Cold-12 h | RPL24 |
| H2O2-6 h | RPL12 | Heat-12 h | RPL26 RPL51 RPL13b |
|  |  | H2O2-12 h | RPL32 RPL37 |

**Supplementary Table 6f: Late (L) genes down-regulation in roots**

| **24 h after treatment** | **Genes downregulated in roots** | **60 h after treatment** | **Genes downregulated in roots** |
| --- | --- | --- | --- |
| Cold-24 h Heat-24 h MeJa-24 h SA-24 h | RPL13a | Cold-60 h MeJa-60 h SA-60 h | RPL13a RPL18a |
| H2O2-24 h Heat-24 h MeJa-24 h SA-24 h | RPL29 RPL4 | H2O2-60 h MeJa-60 h SA-60 h | RPL29 |
| Cold-24 h H2O2-24 h Heat-24 h MeJa-24 h | RPL44 | Cold-60 h H2O2-60 h MeJa-60 h | RPL44 |
| Heat-24 h MeJa-24 h SA-24 h | RPL30 | MeJa-60 h SA-60 h | RPL30 |
| H2O2-24 h MeJa-24 h SA-24 h | RPL6 | Cold-60 h MeJa-60 h | RPL34 RPL22 |
| Cold-24 h Heat-24 h MeJa-24 h | RPL34 | H2O2-60 h MeJa-60 h | RPL5 RPL11 RPL4 RPL15 RPL6 |
| H2O2-24 h Heat-24 h MeJa-24 h | RPL11 RPL5 | Cold-60 h SA-60 h | RPL24b |
| Cold-24 h Heat-24 h SA-24 h | RPL24b RPL18a | Cold-60 h H2O2-60 h | RPL31 RPL21.2 RPL8 RPL10 RPL13b RPL12 RPL7 |
| Cold-24 h H2O2-24 h Heat-24 h | RPL8 RPL10 | MeJa-60 h | RPL28 RPL51 RPL27 |
| Cold-24 h MeJa-24 h | RPL22 | Cold-60 h | RPL14 RPL36 RPL24 |
| Heat-24 h MeJa-24 h | RPL27 RPL28 | H2O2-60 h | RPL19.3 RPL18P RPL23 |
| H2O2-24 h MeJa-24 h | RPL15 |  |  |
| Cold-24 h Heat-24 h | RPL24 RPL26 |  |  |
| Cold-24 h H2O2-24 h | RPL19.3 RPL12 |  |  |
| H2O2-24 h Heat-24 h | RPL31 RPL13b RPL21.2 |  |  |
| Heat-24 h | RPL35 RPL14 RPL36 RPL38 RPL51 RPL32 |  |  |
| H2O2-24 h | RPL18P RPL23 RPL7 |  |  |
